# Vegetation on mesic loamy and sandy soils along a 1700‐km maritime Eurasia Arctic Transect

**DOI:** 10.1111/avsc.12401

**Published:** 2019-02-27

**Authors:** Donald A. Walker, Howard E. Epstein, Jozef Šibík, Uma Bhatt, Vladimir E. Romanovsky, Amy L. Breen, Silvia Chasníková, Ronald Daanen, Lisa A. Druckenmiller, Ksenia Ermokhina, Bruce C. Forbes, Gerald V. Frost, Jozsef Geml, Elina Kaärlejarvi, Olga Khitun, Artem Khomutov, Timo Kumpula, Patrick Kuss, Georgy Matyshak, Natalya Moskalenko, Pavel Orekhov, Jana Peirce, Martha K. Raynolds, Ina Timling

**Affiliations:** ^1^ Alaska Geobotany Center Institute of Arctic Biology & Department of Biology and Wildlife University of Alaska Fairbanks Alaska; ^2^ Department of Environmental Sciences University of Virginia Charlottesville Virginia; ^3^ Plant Science and Biodiversity Center Slovak Academy of Sciences Institute of Botany Bratislava Slovak Republic; ^4^ Geophysical Institute & Department of Atmospheric Science University of Alaska Fairbanks Alaska; ^5^ International Arctic Research Center University of Alaska Fairbanks Alaska; ^6^ Division of Geological & Geophysical Surveys Fairbanks Alaska; ^7^ Earth Cryosphere Institute Tyumen Scientific Center Russian Academy of Sciences, Siberian Branch Tyumen Russia; ^8^ A.N. Severtsov Institute of Ecology and Evolution Russian Academy of Science Moscow Russia; ^9^ Arctic Center University of Lapland Rovaniemi Finland; ^10^ Alaska Biological Research, Inc. Fairbanks Alaska; ^11^ Naturalis Biodiversity Center CR Leiden The Netherlands; ^12^ Department of Ecology and Environmental Sciences Umeå University Umeå Sweden; ^13^ Komarov Botanical Institute Russian Academy of Sciences St. Petersburg Russia; ^14^ University of Tyumen Tyumen Russia; ^15^ University of Eastern Finland Joensuu Finland; ^16^ Institute of Systematic and Evolutionary Botany University of Zürich Zürich Switzerland; ^17^ Department of Soil Science Lomonosov Moscow State University Moscow Russia

**Keywords:** above‐ground biomass ordination, Arctic, bioclimate subzones, Braun‐Blanquet classification, DCA ordination, Normalized Difference Vegetation Index, plant growth forms, remote sensing, soil texture, summer warmth index, tundra biome

## Abstract

**Questions:**

How do plant communities on zonal loamy vs. sandy soils vary across the full maritime Arctic bioclimate gradient? How are plant communities of these areas related to existing vegetation units of the European Vegetation Classification? What are the main environmental factors controlling transitions of vegetation along the bioclimate gradient?

**Location:**

1700‐km Eurasia Arctic Transect (EAT), Yamal Peninsula and Franz Josef Land (FJL), Russia.

**Methods:**

The Braun‐Blanquet approach was used to sample mesic loamy and sandy plots on 14 total study sites at six locations, one in each of the five Arctic bioclimate subzones and the forest–tundra transition. Trends in soil factors, cover of plant growth forms (PGFs) and species diversity were examined along the summer warmth index (SWI) gradient and on loamy and sandy soils. Classification and ordination were used to group the plots and to test relationships between vegetation and environmental factors.

**Results:**

Clear, mostly non‐linear, trends occurred for soil factors, vegetation structure and species diversity along the climate gradient. Cluster analysis revealed seven groups with clear relationships to subzone and soil texture. Clusters at the ends of the bioclimate gradient (forest–tundra and polar desert) had many highly diagnostic taxa, whereas clusters from the Yamal Peninsula had only a few. Axis 1 of a DCA was strongly correlated with latitude and summer warmth; Axis 2 was strongly correlated with soil moisture, percentage sand and landscape age.

**Conclusions:**

Summer temperature and soil texture have clear effects on tundra canopy structure and species composition, with consequences for ecosystem properties. Each layer of the plant canopy has a distinct region of peak abundance along the bioclimate gradient. The major vegetation types are weakly aligned with described classes of the European Vegetation Checklist, indicating a continuous floristic gradient rather than distinct subzone regions. The study provides ground‐based vegetation data for satellite‐based interpretations of the western maritime Eurasian Arctic, and the first vegetation data from Hayes Island, Franz Josef Land, which is strongly separated geographically and floristically from the rest of the gradient and most susceptible to on‐going climate change.

**Nomenclature**
Pan‐Arctic Species List (PASL) (Raynolds et al., 2013), a circumpolar compendium of accepted names for vascular plants (Elven, Murray, Razzhivin, & Yurtsev, 2011), mosses (Belland et al., 2012), liverworts (Konstantinova & Bakalin, 2009) and lichens (Kristinsson, Hansen, & Zhurbenko, 2010). 

## 1 INTRODUCTION

Arctic tundra ecosystems occur in a broad circumpolar belt that extends from areas north of 80°N to forest–tundra areas south of 60°N, with mean July temperatures that vary from near 0°C to over 12°C. Several conceptual approaches have been used to subdivide the vegetation along the broad bioclimate gradients of Eurasia (Alexandrova, 1980; Chernov & Matveyeva, 1997; Yurtsev, 1994a), North America (Bliss, 1997; Daniëls, Bültmann, Lünterbusch, & Wilhelm, 2000; Edlund, 1990; Polunin, 1951) and the circumpolar Arctic (Elvebakk, Elven, & Razzhivin, 1999; Tuhkanen, 1984; Walker et al., 2005; Yurtsev, 1994b). Only a few studies, however, have attempted to examine continuous vegetation transitions of zonal plant communities along transects that traverse the full Arctic bioclimate gradient because of the rather daunting logistics involved. Examples exist for the Taymyr Peninsula, Russia (Matveyeva, 1998), the North America Arctic Transect (NAAT; Walker, Kuss, et al., 2011) and the 1999 Canada transect for the Circumpolar Arctic Vegetation Map (Gonzalez, Gould, & Raynolds, 2000). Arctic alpine vegetation gradients have been described along elevation gradients in the mountains of southwest Greenland (Sieg, Drees, & Daniëls, 2006).

**Figure 1:**
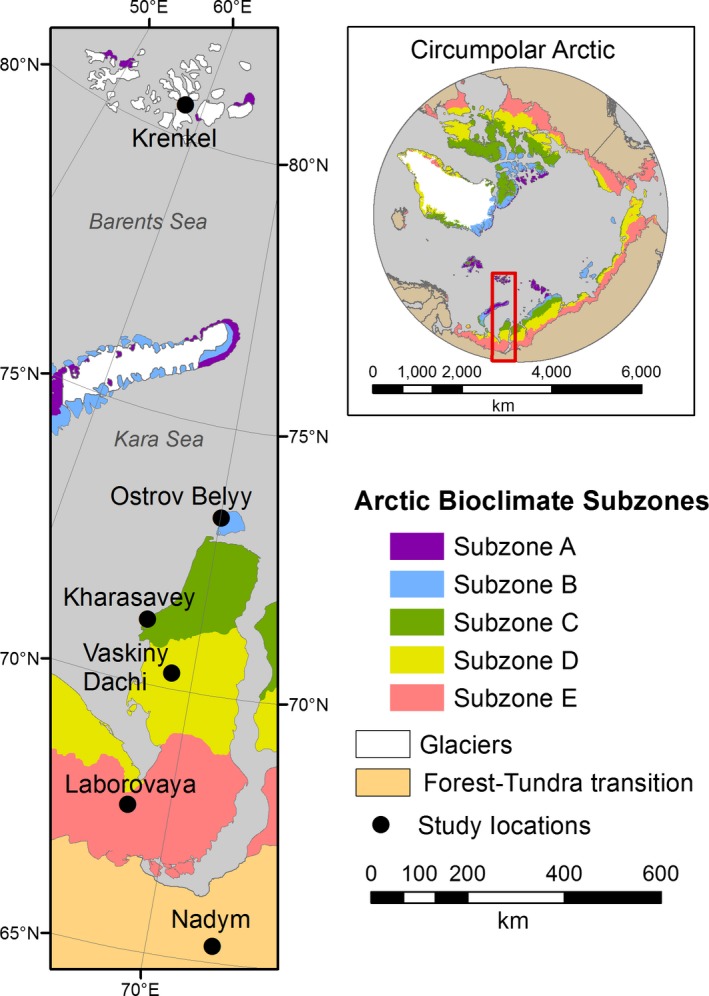
The Eurasia Arctic Transect and Arctic bioclimate subzones. Inset map shows circumpolar distribution of the subzones according to the Circumpolar Arctic Vegetation Map (CAVM Team et al., 2003)

Here we describe the vegetation along the 1700‐km Eurasia Arctic Transect (EAT) that includes the Yamal Peninsula and Franz Josef Land (Figure 1). The aim is to characterize vegetation on zonal loamy and sandy soils along the complete maritime Arctic climate gradient in western arctic Russia to aid in remote‐sensing interpretations of land‐cover and land‐use change (Walker, Epstein, et al., 2012). The zonal patterns, geological conditions, permafrost and summer thaw depth (active layer) conditions are generally well described along the length of the peninsula. We analyse the variations in plant growth forms and species richness in each layer of the plant canopy with respect to summer temperature and soil texture, present a preliminary numerical classification and use indirect ordination methods to analyse the relationship of the plots and species to a suite of measured environmental factors.

## 2 METHODS

### 2.1 Site selection and sampling

We established the EAT during four expeditions in the summers of 2007–2010 (Figure 1). The transect extends from the Krenkel Hydro‐meteorological Station on Hayes Island (80°37′N, 58°03′E) in the maritime polar desert of Franz Josef Land, to Nadym (65°19′N, 72°53′E) in the forest–tundra transition of west Siberia. Mean July temperatures range from 1°C at the northern end of the transect to 15.8°C at the southern end. Six study locations were selected along the EAT to represent zonal (Razzhivin, 1999; Walter, 1954, 1973) vegetation conditions in each of the five Arctic bioclimate subzones and the forest–tundra transition, as mapped on the Circumpolar Arctic Vegetation Map (Walker et al., 2005; Yurtsev, 1994b; Table 1). At each location we chose at least two study sites — one on mesic loamy soils and one on mesic sandy soils (see Supporting Information Appendix S1 for geological setting in relationship to soils).

**Table 1:** Study locations, site numbers, site names, microsites, geological settings, parent material, and dominant vegetation at each study site

Location	Coordinates	Bioclimate subzone	Site	Geological setting^a^, parent material	Microsite	Plot field numbers	Dominant vegetation
Krenkel	80°37′N, 58°03′E	A	KR‐1, Loamy	Deluvial slope, perhaps old marine terrace at 30 m, sands		KR_RV_60–64	*Papaver dahlianum* spp*. polare, Stellaria edwardsii, Cetrariella delisei, Ditrichum flexicaule,* biological soil crust, cushion‐forb, lichen, moss tundra
			Kr‐2 Sandy	Recent marine terrace at 10 m, marine sands		KR_RV_65–69	*Papaver dahlianum* spp*. polare, Stellaria edwardsii, Cetrariella delisei*, biological soil crust, cushion‐forb, lichen, moss tundra
Ostrov Belyy	73°19′N, 70°03′E	B	OB‐1, loamy	Marine terrace II, alluvial‐marine sediments, loamy facie of mixed sands and silts	OB‐1a, Non‐sorted circles	OB_RV_49a–53a	*Carex bigelowii, Calamagrostis holmii, Salix polaris, Hylocomium splendens,* graminoid, prostrate‐dwarf‐shrub, moss tundra
					OB‐1b, Inter‐circle areas	OB_RV_49b–53b	*Dryas integrifolia, Arctagrostis latifolia, Racomitrium lanuginosum, Ochrolechia frigida,* prostrate‐dwarf‐shrub, crustose‐lichen barren
			OB‐2, Sandy	Marine terrace I, alluvial‐marine sediments, sands	OB‐2a, Small non‐sorted‐polygon centres	OB_RV_54a–58a	*Gymnomitrion corallioides‐Salix nummularia‐Luzula confusa‐Ochrolechia frigida,* liverwort, prostrate‐dwarf‐shrub, graminoid, lichen tundra
					OB‐2b, Polygon cracks	OB_RV_53b–58b	*Racomitrium lanuginosum, Salix nummularia,* moss, prostrate‐dwarf‐shrub tundra
Kharasavey	71°12′N, 66°56′E	C	KH‐1, loamy	Marine terrace II, marine silts		KH_RV_40–44	*Carex bigelowii, Calamagrostis holmii, Salix polaris, Dicranum elongatum, Cladonia* spp., graminoid, prostrate‐dwarf‐shrub, moss tundra
			KH‐2a, sandy	Marine terrace I, marine silts		KH_RV_45–46	*Carex bigelowii, Salix nummularia, Dicranum* sp*., Cladonia* spp., graminoid, prostrate‐dwarf‐shrub, moss, lichen tundra
			KH‐2b, sandy	Marine terrace II, marine sands and silts		KH_RV_47–49	*Salix nummularia, Luzula confusa, Polytrichum strictum, Sphaerophorus globosus,* prostrate‐dwarf‐shrub, graminoid, moss, lichen tundra
Vaskiny Dachi	70°17′N, 68°54′E	D	VD‐1, loamy	Coastal marine plain terrace IV,, mixed Alluvial sands and marine silts		VD_RV_25–29	*Carex bigelowii, Vaccinium vitis‐idaea, Hylocomium splendens,* sedge, dwarf shrub, moss tundra
			VD‐2, loamy	Fluvial marine terrace III, mixed alluvial sands and marine silts		VD_RV_30–34	*Betula nana, Calamagrostis holmii, Aulacomnium turgidum,* erect‐dwarf‐shrub, graminoid, moss tundra
			VD‐3, sandy	Fluvial terrace II, alluvial and aeolian reworked sands		VD_RV_35–39	*Vaccinium vitis‐idaea, Cladonia arbuscula, Racomitrium lanuginosum,* prostrate‐dwarf‐shrub, sedge, lichen, tundra
Laborovaya	67°42′N, 68°01′E	E	LA‐1, loamy	Glacial terrace, glacial silt		LA_RV_15–19	*Carex bigelowii, Betula nana, Aulacomnium palustre,* sedge, erect‐dwarf‐shrub, moss tundra
			LA‐2, sandy	Recent (<10 kya) alluvial terrace of stream, alluvial sand		LA_RV_20–21	*Betula nana, Vaccinium vitis‐idaea, Sphaerophorus globosus, Polytrichum strictum,* prostrate‐dwarf‐shrub, lichen tundra
Nadym	65°19′N, 72°53′E	Forest–tundra transition	ND‐1, loamy, forest	Fluvial terrace II, alluvial loamy sands		ND_RV_01–05	*Pinus sylvestris, Betula tortuosa, Rhododendron tomentosum, Cladonia stellaris,* erect‐dwarf‐shrub, lichen woodland
			ND‐2, sandy, tundra	Fluvial terrace III, alluvial sands	ND‐2a, Hummocks	ND_RV_06–08	*Rhododendron tomentosum, Betula nana, Cladonia stellaris,* erect‐dwarf‐shrub, lichen tundra
					ND‐2b, Interhummocks	ND_RV_09–11	*Cladonia stellaris, Carex glomerata,* lichen tundra

a Marine and alluvial terrace numnbers (see Supporting Information Appendix S1), approximate elevations above mean sea level on the Yamal Peninsula, approximate ages: Marine terrace I, 7–12 m a.s.l., Sartansky‐age (Last Glacial Maximum, Late Wiechselian), ≈10–25 ka; Marine terrace II, 10–25 m a.s.l., Karginsky‐Zyransky‐age (Middle Weichselian), ≈25–75 ka; Marine terrace III, 26–40 m a.s.l., Ermanovsky‐age (Early Weichselian), ≈75–117 ka; Marine terrace IV, 40–45 m a.s.l., Kazantsevskaya‐age (Eemian interglacial), ≈117–130 ka; Marine terrace V, 45–58 m a.s.l., Salekhardskaya age (Saalian), ≈130–200 ka.

We used the Braun‐Blanquet approach (Westhoff & Van der Maarel, 1978) to sample mesic loamy and sandy sites at each location. At most study sites there was adequate space for a large relatively homogeneous 50 m × 50 m sample site that corresponded approximately to the 30‐m to 70‐m pixel size of the Landsat satellite sensors. Sample plots and transects were arranged in the pattern shown in Supporting Information Appendix S2. Here we describe the data mainly from 5 m × 5 m (25 m^2^) plots, except at the Nadym forest site, where 10 m × 10 m (100 m^2^) plots were used, and the Nadym tundra site, where 1 m × 1 m (1 m^2^) plots were used to sample homogeneous areas of vegetation on patterned ground features (earth hummocks). We sampled 79 plots, but eliminated three Nadym wetland plots, resulting in a final data set of 76 plots, distributed among the six EAT locations: Krenkel (KR, ten plots), Ostrov Belyy (BO, 20 plots), Laborovaya (LA, ten plots), Kharasavey (KH, ten plots), Vaskiny Dachi (VD, 15 plots) and Nadym (ND, 11 plots) (see Supporting Information Appendix S3 for descriptions and photographs of the study sites.)

Each vascular plant, bryophyte and lichen species occurring within a plot was recorded and a sample taken as a voucher. Unknown species were sent to the Komarov Botanical Institute (KBI) for final identification. The cover‐abundance of each species was recorded using Braun‐Blanquet categories (*r* = single occurrence; + = several occurrences but <1% cover; 1 = 1%–5% cover; 2 = 6%–25%; 3 = 26%–50%; 4 = 51%–75%; 5 = 76%–100%; Braun‐Blanquet, 1928). For calculating the mean cover, the cover‐abundance scores were transformed to a mean percentage score corresponding to the midpoint of each cover‐abundance category: r = 0.05; + = 0.5; 1 = 2.5; 2 = 15.0; 3 = 37.5; 4 = 62.5; 5 = 87.5. Plant species were also assigned to plant growth form (PGF) categories (Supporting Information Appendix S4).

The environmental data from each plot include 107 variables, including site, soil, biomass, spectral data, NDVI and canopy structure variables. (see details in, Supporting Information Appendices S5.1 and S5.2, and the project data reports; Walker, Carlson, et al., 2011; Walker, Epstein, et al., 2008; Walker, Epstein, et al., 2009; Walker, Orekhov, et al., 2009).

Soils samples were collected from the uppermost mineral soil horizons at a point just outside the southwest corner of each vegetation plot. Larger soil pits were dug just outside the southwest corner of the 50 m × 50 m grid to fully describe vertical and horizontal variation in the soil profiles. The pits were described by Dr. Georgy Matyshak according the Russian approach and translated into descriptions corresponding to the US Soil Taxonomy approach (Soil Survey Staff, 1999) and are included with photographs in the data reports cited above.

### 2.2 Climate

The Arctic bioclimate zonation patterns portrayed on the Circumpolar Arctic Vegetation Map (CAVM Team et al., 2003) are based primarily on summer temperature regimes and structure of the vegetation (Yurtsev, Tolmachev, & Rebristaya, 1978; Yurtsev, 1994a). We use the summer warmth index (SWI), which is the sum of monthly mean temperatures above 0°C, measured in °C month “thawing degree months”. The SWI is calculated from monthly mean temperature data and is very strongly correlated with thawing degree days, which require daily mean temperature to calculate. SWI is equivalent to the warmth index, *a*, used by Steve Young for the vascular plant flora of St. Lawrence Island, Alaska (Young, 1971). Four of the six EAT locations have long‐term climate station data; for these locations, we calculated the SWI for air temperatures (SWI_a_) at the standard 2 m height of weather station observations. To obtain consistent summer temperature data for all study locations over the same length of record, we used data from the thermal infrared channels of satellite‐based Advanced Very High Resolution Radiometers (AVHRR, years 1982–2003; Comiso, 2003, 2006) to calculate SWI_g_, the ground surface summer warmth index (SWI_g_) within 12.5‐km pixels containing the study locations (Bhatt et al., 2010). Consistent data for other climate factors, such as precipitation and wind, were not available across all study locations.

### 2.3 Vegetation analysis

#### 2.3.1 Cluster analysis

We used a hierarchical dendrogram approach, available in PC‐ORD to group the plots into clusters based on the similarity of their species compositions (MjM Software, Gleneden Beach, OR, US) via the JUICE 7.0 software (Tichý, 2002). The most meaningful separation of the 76 plots was achieved with the flexible beta group linkage method (β = −0.25) with the Sørensen distance measure and square root data transformation. We included species‐level taxonomic determinations in the analyses, and we excluded taxa that were identified only to the genus level. To determine the optimal number of clusters providing the highest ‘separation power’ for the data set, we used the Crispness of Classification approach (Botta‐Dukát, Chytrý, & Hájková, 2005) available through the Optimclass function in JUICE (Tichý, 2002). A synoptic table was prepared using the combined synoptic table function in JUICE. Taxa with high fidelity (modified phi coefficients ≥ 0.5) were interpreted as diagnostic for the group; taxa with very high fidelity (modified phi coefficients ≥ 0.8) were interpreted as highly diagnostic.

#### 2.3.2 Analysis of vegetation and environmental variables

We compared the trends of plant growth form (PGF) cover along the bioclimate gradient (SWI_g_) for each layer of the plant canopy (tree and shrub layer, herb layer and cryptogam layer); and the species richness within groups of dominant PGFs (deciduous shrubs, evergreen shrubs, graminoids, forbs, mosses, lichens). We also examined trends of soil properties along the bioclimate gradient.

#### 2.3.3 Ordination

We explored several ordination methods available in the R program (R Foundation for Statistical Computing, Vienna, AT) through the JUICE vegetation analysis package (Tichý, 2002). Detrended Correspondence Analysis (DCA; Hill & Gauch, 1980) provided the clearest, most easily interpreted separation of plots along complex environmental gradients. Plot and species similarities were calculated using the Sørenson similarity index. Rare species were down‐weighted and the axes scaled according to the program defaults. The four main DCA axes 1, 2, 3 and 4 were correlated with continuous and ordinal environmental variables in each plot using species–environment correlations in the program CONOCO via JUICE. Only variables with *p *≤ 0.002 determined by global permutation test with forward selection (number of permutations: 499) are shown in the biplot diagrams.

## 3 RESULTS

### 3.1 Descriptions of the EAT locations and study sites

An overview of the study sites (Table 1) includes the study locations, coordinates, bioclimate subzones, study site numbers, geological setting, parent material, field plot numbers and dominant vegetation. Descriptions and photos of the environment and vegetation of each study location and study site are in Supporting Information Appendix S3. The species and environmental data from the 79 sample plots are in Supporting Information Appendices S4 and S5.

Mean July temperatures range from 1°C at Krenkel to 15.8°C at Nadym. Mean annual precipitation ranges from 258 mm at Ostrov Belyy to 479 mm at Nadym (Table 2). The SWI_g_ values at the EAT study locations are generally within one *SD* of the circumpolar SWI_g_ means of bioclimate subzones B to E (Table 2, columns 6 and 7), which indicates that these locations are representative of the mean zonal summer temperature conditions. The exception is Krenkel (SWI_g_ = 2°C month), which is much colder than the mean SWI_g_ for subzone A (8.2 ± 3.4°C month). The 12.5 km pixels of the satellite‐derived SWI_g_ are subject to subpixel effects arising from the contrasting temperature regimes of different surfaces, especially near glaciers and coastlines (Smith, Reynolds, Peterson, & Lawrimore, 2008); however, the satellite‐derived SWI_g_ values are within 1°C month of the station SWI_a_ values at all EAT study locations where station data are available, including the three coastal locations, (Table 2, columns 5 and 3).

**Table 2:** Temperature and precipitation along the Eurasia Arctic Transect. Mean (1961–1990) July temperature and precipitation data (columns 3 and 4) are from the nearest relevant climate stations. Summer Warmth Index (SWI) is the sum of the monthly mean temperatures above freezing. The mean atmospheric SWI (SWI_a_) (column 5) is calculated from the mean (1961–1990) station data, where available. Ground Summer Warmth Indices (SWIg) (column 6) are calculated from AVHRR thermal bands for the 12.5‐km pixels containing the EAT study locations. Value for SWI_g_ in the circumpolar Arctic subzones (column 7) are calculated using all circumpolar pixels within each subzone (Raynolds et al., 2008)

Bioclimate subzone	EAT study location	Mean July Temp. (1961–1990, °C)^a^	Mean annual precipitation (1961–1990, mm)^a^	Mean SWI_a_ at local climate station (1961–1990, °C month)^a^	Mean SWI_g_ for 12.5‐km pixel containing the location (°C month)	Mean SWI_g_ for Circum‐polar Arctic subzones (Mean ± *SD* °C month)
A	Krenkel	1	282	1.1	2.0	8.2 ± 3.4
B	Ostrov Belyy	5.6	258	11	11.5	12.6 ± 5.8
C	Kharasavey	7.2^b^	310^b^	18.6^b^	18.5	19.8 ± 5.1
D	Vaskiny Dachi	ND	ND	ND	29.6	27.0 ± 4.9
E	Laborovaya	ND	ND	ND	36.6	33.2 ± 4.4
FT‐transition	Nadym	15.8	479	43	41.3	ND

a Leibman et al. (2012).

b Data from Mare Sale, closest coastal station to Kharasavey, 100 km south.

**Figure 2:**
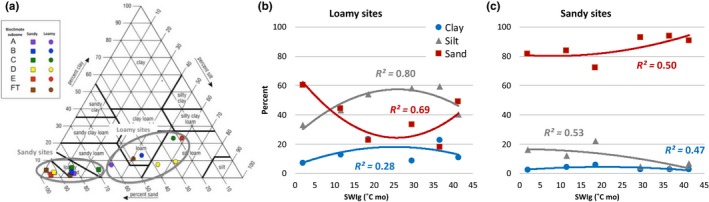
Mean soil textures for EAT loamy sites and sandy sites. (a) Mean soil texture classes for each site plotted on a USDA soil texture triangular (percentage sand, silt, clay) with 12 size classes defined by the US Department of Agriculture (Soil Survey Staff, 1999). Each point represents the mean of five plots except for the FT‐sandy (brown squares), which portray mean values for hummocks (loamy sand) and inter‐hummock (sand) plots. (b) Sand, silt and clay percentages at loamy sites vs. summer warmth index (SWI_g_). (c) Sand, silt and clay percentages at sandy sites vs. summer warmth index (SWI_g_). Best‐fit regression equations are in Supplemental Information Appendix 9

Clay, silt and sand percentages for loamy and sandy sites are shown using the US Department of Agriculture soil texture triangle (Figure 2a). Loamy sites had 19%–61% sand and 31%–62% silt. Sandy sites generally had >80% sand, and <20% silt. Clay percentages were low (<25%) at all sites. On the loamy sites, silt and clay percentage were somewhat higher in the central part of the summer temperature gradient. Sand percentages were higher at both ends of the gradient (Figure 2b).

### 3.2 Classification and syntaxonomic interpretation

**Figure 3:**
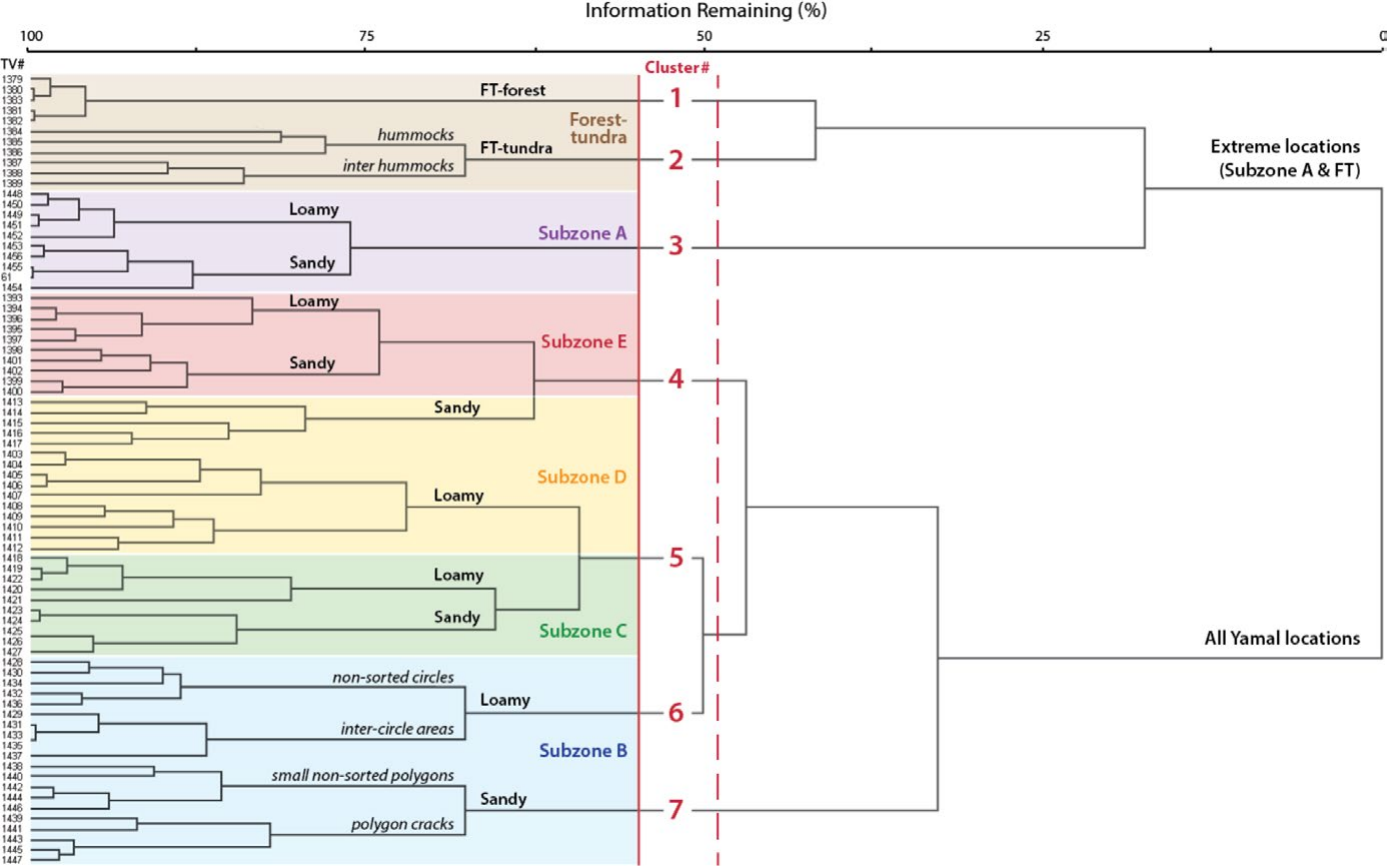
Cluster analysis of EAT plots. The plot is based on similarity of species composition within the 76 plots using Sørensen's coefficient of distance measure and square root data transformation. The numbers on the left side of the diagram are consecutive plot numbers assigned in the Turboveg program. Corresponding plot field numbers are in the Supporting Information Appendix S3. All species (vascular plants, bryophytes and lichens) were included. Plots linked toward the left side of the diagram have high species similarity; linkages toward the right side of the diagram have low levels of similarity. The flexible‐β group linkage method (β = −0.25) was used to hierarchically link the plots. The vertical red dashed line shows the second optimal level of clustering based the Crispness of Classification approach (Botta‐Dukát et al., 2005) available through the Optimclass function in JUICE (Tichý, 2002), which resulted in the six optimal clusters (red numbers). The red line is where the line was adjusted to separate out cluster 6, which based on field observations was distinct from cluster 5. Background colours correspond to the bioclimate subzones (A to Forest–tundra). Also shown are loamy and sandy groups of plots (black Roman labels), and micro‐topographic groups of plots in patterned ground complexes (italics)

The cluster analysis dendrogram shows the progressive linkage of plots according to their floristic similarity (Figure 3). Clusters with higher levels of similarity are toward the left side of the diagram. Crispness of Classification identified two clusters with the highest level of separability (dissimilarity). One cluster contained all of the Yamal plots (subzones B, C, D and E) and the other contained all the plots of FJL (subzone A) and Nadym (FT transition). The next highest level of dissimilarity was achieved with six clusters, separated at the level of the red dashed line in Figure 3. At this level, clusters 5 and 6 in Figure 3 were joined, forming one large cluster containing most of the plots on the Yamal Peninsula, including the subzone D loamy plots, all subzone C plots and the subzone B loamy plots. Based on our knowledge of the rather unique floristic character of the loamy subzone B site, which has characteristics similar to the moist non‐acidic tundra described from North America, Greenland and Russia, we shifted the breakpoint for cluster definition slightly to the left so that the subzone B loamy plots were recognized as a separate cluster, resulting in a final grouping with seven clusters.

A synoptic table (Table 3) shows the frequency of species with very high fidelity (modified phi ≥ 0.8) and high fidelity (0.8 > modified phi ≥ 0.5). The full synoptic table, including diagnostic and non‐diagnostic taxa, is in Supporting Information Appendix S6. Lists of the diagnostic, frequent and dominant taxa in each cluster are in Supporting Information Appendix S7. A summary of the contents of the clusters and their alignment with described Br.‐Bl. syntaxa (mostly classes) are as follows:

**Table 3:** Synoptic table containing diagnostic taxa for statistical clusters of mesic tundra vegetation plots along the Eurasia Arctic Transect

Cluster no.		1	2	4	5	6	7	3
Subzone(s) (soil texture)		FT(lom)	FT(snd)	E+D(snd)	D(lom)+C	B(lom)	B(snd)	A
Number of plots		5	6	15	20	10	10	10
Diagnostic taxa for cluster 1	Growth form			.	.	.	.	.
*Pinus sylvestris*	tne	100	.	.	.	.	.	.
*Betula pubescens*	tbd	100	.	.	.	.	.	.
*Larix sibirica*	tnd	100	.	.	.	.	.	.
*Vaccinium myrtillus*	sdd	100	.	.	.	.	.	.
*Juniperus communis*	sle	80	.	.	.	.	.	.
*Peltigera malacea*	lfo	60	.	.	.	.	.	.
*Pleurozium schreberi*	bmp	100	17	47	5	.	.	.
*Peltigera leucophlebia*	lfo	100	.	13	50	20	.	.
*Cladonia stellaris*	lfr	100	83	20	.	.	.	.
*Empetrum nigrum*	sde	100	17	80	10	.	.	.
*Vaccinium uliginosum*	sdd	100	33	67	15	.	.	.
Diagnostic taxa for cluster 2								
*Carex globularis*	gs	.	100	.	.	.	.	.
*Andromeda polifolia*	sde	.	83	7	.	.	.	.
*Rubus chamaemorus*	sdd	.	83	7	.	.	.	.
*Rhododendron tomentosum* s. *tomentosum*	sle	100	100	73	.	.	.	.
Diagnostic taxa for cluster 4								
*Flavocetraria nivalis*	lfr	.	.	93	25	.	.	.
*Salix phylicifolia*	sld	.	.	67	10	.	.	.
*Eriophorum vaginatum*	gs	.	17	87	25	.	.	.
*Pedicularis labradorica*	fe	.	.	53	.	.	.	.
*Asahinea chrysantha*	lfr	.	.	40	.	.	.	.
*Pertusaria dactylina*	lc	.	.	47	.	.	10	.
*Cladonia grayi*	lfr	.	.	40	5	.	.	.
*Schljakovia kunzeana*	bl	.	.	33	.	.	.	.
*Luzula wahlenbergii*	gr	.	.	33	.	.	.	.
Diagnostic taxon for clusters 5 & 6								
*Arctagrostis latifolia*	gg	.	.	20	95	100	10	.
Diagnostic taxa for cluster 5								
*Lophozia ventricosa*	bl	.	.	40	80	.	.	.
*Alopecurus borealis*	gg	.	.	.	60	.	.	10
*Salix reptans*	sdd	.	.	13	55	.	.	.
*Eriophorum angustifolium*	gs	.	.	27	60	.	.	.
*Tephroseris atropurpurea*	fe	.	.	7	45	.	.	.
*Peltigera canina*	lfo	.	.	.	35	.	.	.
*Peltigera aphthosa*	lfo	.	.	.	40	10	.	.
*Lichenomphalia hudsoniana*	lfo	.	.	.	30	.	.	.
Diagnostic taxa for cluster 6								
*Blepharostoma trichophyllum*	bl	.	.	.	5	100	.	.
*Salix polaris*	sdd	.	.	.	50	100	.	.
*Tomentypnum nitens*	bmp	.	.	13	20	90	.	.
*Dryas octopetala*	sde	.	.	.	40	100	50	.
*Poa arctica*	gg	.	.	7	40	80	.	.
*Juncus biglumis*	gr	.	.	.	.	60	20	.
*Bryum cyclophyllum*	bma	.	.	.	.	40	.	.
*Stellaria longipes*	fe	.	.	.	25	60	.	.
*Sphenolobus minutus*	bl	.	.	73	80	100	20	.
Diagnostic taxa for cluster 7								
*Pogonatum dentatum*	bma	.	.	13	.	.	80	.
*Oxyria digyna*	fm	.	.	.	.	.	80	20
*Gymnomitrion corallioides*	bl	.	.	33	25	10	100	.
*Luzula confusa*	gr	.	.	.	60	10	100	.
*Salix nummularia*	sdd	.	.	27	50	.	100	.
*Lloydia serotina*	fe	.	.	.	.	.	50	.
*Solorina crocea*	lfo	.	.	.	.	.	50	.
*Polytrichum piliferum*	bma	.	.	7	.	10	50	.
*Pohlia crudoides*	bma	.	.	7	.	.	40	.
*Gowardia nigricans*	lfr	.	.	40	60	20	90	.
Diagnostic taxa for cluster 3								
*Stellaria longipes* taxon *edwardsii*	fe	.	.	.	.	.	.	100
*Papaver dahlianum* agg. (*P. cornwallisense*)	fm	.	.	.	.	.	.	100
*Phippsia algida*	gg	.	.	.	.	.	.	100
*Cochlearia groenlandica*	fm	.	.	.	.	.	.	100
*Lecidea ramulosa*	lc	.	.	.	.	.	.	100
*Orthothecium chryseum*	bmp	.	.	.	.	10	.	100
*Cladonia pocillum*	lfr	.	.	.	.	10	.	100
*Cetrariella delisei*	lfr	.	.	20	.	.	.	100
*Cerastium nigrescens* v*. laxum*	fm	.	.	.	.	.	.	80
*Fulgensia bracteata*	lc	.	.	.	.	.	.	80
*Saxifraga cernua*	fe	.	.	.	5	.	.	80
*Draba subcapitata*	fm	.	.	.	.	.	20	90
*Cirriphyllum cirrosum*	bmp	.	.	.	.	.	.	70
*Cerastium regelii*	fm	.	.	.	.	10	.	70
*Encalypta alpina*	bma	.	.	.	.	.	.	60
*Solorina bispora*	lfo	.	.	.	.	.	.	60
*Bryum rutilans*	bma	.	.	.	.	.	.	60
*Saxifraga cespitosa*	fm	.	.	.	.	.	.	60
*Distichium capillaceum*	bma	.	.	.	.	30	.	80
*Cetraria aculeata*	lfr	.	.	.	.	.	20	70
*Pohlia cruda*	bma	.	.	.	.	40	.	80
*Gowardia arctica*	lfr	.	.	.	.	.	.	50
*Saxifraga oppositifolia*	fm	.	.	.	.	.	.	50
*Cladonia symphycarpia*	lfr	.	.	.	.	.	.	50
*Stereocaulon rivulorum*	lfr	.	.	.	.	.	.	50
*Polytrichastrum alpinum*	bma	.	.	.	30	10	60	100
*Bartramia ithyphylla*	bma	.	.	.	.	.	10	50
*Callialaria curvicaulis*	bmp	.	.	.	.	.	.	40
*Campylium stellatum* v. *arcticum*	bmp	.	.	.	.	.	.	40
*Ditrichum flexicaule*	bma	.	.	.	5	40	.	70
*Protopannaria pezizoides*	lc	.	.	.	5	.	.	40

Notes

Values are frequency of the given plant taxon within the indicated cluster (see Figure 3). Fidelity of diagnostic species was calculated using the phi coefficient (Chytrý, Tichý, Holt, & Botta‐Dukát, 2002) for individual clusters compared to the full suite of clusters. Diagnostic taxa are ordered according to descending fidelity (modified phi values). Taxa with very high fidelity (modified phi ≥ 0.8) have frequency values highlighted in dark grey; those with high fidelity (modified phi ≥ 0.5) are highlighted in light grey. The second column in the table contains the plant growth form for each species: bl, bryophyte, liverwort; bma, bryophyte, moss, acrocarpous; bmp, bryophyte, moss, pleurocarpous; bms, bryophyte, moss, sphagnoid; fe, forb, erect; fm, forb, mat, cushion or rosette; gs, graminoid, sedge; gg, graminoid, grass; gr, graminoid, rush; lc, lichen, crustose; lfo, lichen, foliose; lfr, lichen, fruticose; sle, shrub, low, evergreen; sld, shrub, low, deciduous; sde, shrub, dwarf, evergreen; sdd, shrub, dwarf, deciduous; tne, tree, needle‐leaf, evergreen; tnd, tree, needle‐leaf, deciduous; tbd, tree, broad‐leaf, deciduous; vs, vascular plant, seedless. A dot (.) indicates no record of the indicated species in the indicated cluster.

*Cluster 1* contains the five forest plots at Nadym with five highly diagnostic taxa (phi ≥ 0.8; *Pinus sylvestris, Betula pubescens, Larix sibirica, Vaccinium myrtillus, Juniperus communis*) and six other diagnostic taxa (phi ≥ 0.5). This cluster aligns with Cl. *Vaccinio–Piceetea* and All. *Vaccinio uliginosi–Pinion sylvestris* Br.‐Bl (Braun‐Blanquet). in Br.‐Bl. et al. 1939, which contains Holarctic coniferous and boreo‐subarctic birch forests on oligotrophic and leached soils in the boreal zone (Mucina et al., 2016).
*Cluster 2* contains the six tundra plots in the forest–tundra transition at Nadym with three highly diagnostic taxa (*Carex globularis, Andromeda polifolia, Rubus chamaemorus*) and one other diagnostic taxon (*Rhododendron tomentosum*) This cluster aligns with Cl. *Oxycocco–Sphagnetea* Br.‐Bl. et Tx. ex Westhoff et al. 1946, which contains dwarf shrub, sedge and peat moss vegetation of the Holarctic ombrotrophic bogs and wet heaths on extremely acidic soils.
*Cluster 3* contains all ten plots in subzone A at Krenkel. This is the most distinctive cluster with 13 highly diagnostic taxa (*Stellaria edwardsii, Papaver dahlianum, Phippsia algida, Cochlearia groenlandica, Lecidea ramulosa, Orthothecium chryseum, Cladonia pocillum, Cetraria delisei*) and 18 other diagnostic taxa. Many of these are diagnostic for the recently described “polar desert” Br.‐Bl. class *Drabo corymbosae–Papaveretea dahlilani* (Daniëls, Elvebakk, Matveyeva, & Mucina, 2016), which contains cushion forb, lichen, moss tundra occurring in polar deserts of the Arctic zone of the Arctic Ocean archipelagos (Mucina et al., 2016).
**Clusters 4, 5, 6 and 7** form a broad group of plots across the central part of the Yamal Peninsula with a general trend from relatively warm sites in cluster 4 (subzones E and D) to relatively cold sites in clusters 6 and 7 (subzone B). Although all four clusters have several diagnostic taxa (phi > 0.5), there are only three highly diagnostic taxa (phi ≥ 0.8) in the group. *Cluster 4* contains the ten subzone E plots at Laborovaya and the five sandy plots in subzone D at Vaskiny Dachi. It has one highly diagnostic taxon (*Flavocetraria nivalis*) and eight other diagnostic taxa. This cluster aligns weakly with Cl. *Oxycocco–Sphagnetea* Br.‐Bl. et Tx. ex Westhoff et al. 1946, which contains dwarf‐shrub, sedge and peat‐moss vegetation of the Holarctic ombrotrophic bogs and wet heaths on extremely acidic soils (Mucina et al., 2016). *Cluster 5* contains the ten subzone D loamy plots and ten subzone C plots. It has eight diagnostic taxa (*Lophozia ventricosa, Alopecurus borealis, Salix reptans, Eriophorum angustifolium*,* Tephroseris atropurpurea, Peltigera canina, P. aphthosa, Lichenomphalia hudsoniana*) and no highly diagnostic taxa. This cluster weakly aligns with Cl. *Scheuchzerio palustris–Caricetea fuscae* Tx. 1937, which contains sedge, moss vegetation of fens, transitional mires and bog hollows in the temperate, boreal and Arctic zones (Mucina et al., 2016). *Cluster 6* contains the five loamy plots at Ostrov Belyy, each of which has two microhabitat subplots corresponding to non‐sorted circles and inter‐circle areas. It has one highly diagnostic taxon (*Blepharostoma trichophyllum*) and eight other diagnostic taxa (*Salix polaris, Tomentypnum nitens, Dryas octopetala, Poa arctica, Juncus biglumis Bryum cyclophyllum, Stellaria longipes, Sphenolobus minutus*). This cluster weakly aligns with Cl. *Carici rupestris‐Kobresietea bellardii* Ohba 1974, which contains, circum‐Arctic fellfield and dwarf‐shrub graminoid tundra on base‐rich substrates (Mucina et al., 2016). It has characteristics of plant communities occurring on moist non‐acidic soils in Alaska [Ass. *Dryado integrifoliae–Caricetum bigelowii* (Walker, Walker, & Auerbach, 1994)], Greenland [*Eriophorum angustifolium–Rhododendron lapponicum* comm. (Lünterbusch & Daniels, 2004)] and the Taimyr Peninsula, Russia [*Carici arctisibiricae–Hylocomietum alaskana* (Matveyeva, 1994)]. *Cluster 7* contains the ten subzone B sandy plots at Belyy Ostrov. It has one highly diagnostic taxon (*Pogonatum dentatum*) and 12 other diagnostic taxa (*Oxyria digyna, Gymnomitrion corallioides, Luzula confusa, Salix nummularia, Lloydia serotina, Solorina crocea, Polytrichum piliferum, Pohlia crudoides, Gowardia nigricans*). This cluster very weakly aligns with Cl. *Saxifrago cernuae–Cochlearietea groenlandicae* Mucina et Daniëls 2016, which contains vegetation of open graminoid tundra disturbed by cryoturbation (Mucina et al., 2016).

### 3.3 Soils, vegetation structure and species richness

Trends of key soil and key vegetation canopy factors (canopy layer height, litter, standing dead, LAI, NDVI, total phytomass) vs. SWI_g_ are in Supporting Information Appendix S8.

Soil properties that increase with higher SWI_g_ include percentage sand (on sandy sites), thickness of organic horizons, percentage soil carbon (on loamy sites) and active layer thickness (Supporting Information Appendix S8, Figure S8‐1). Soil properties that tend to decrease with SWI_g_ include soil pH, soil moisture and sodium concentration. Loamy sites have generally higher volumetric soil moisture, pH, cation exchange capacity (CEC), sodium, volumetric soil moisture, thicker organic soil horizons, more soil carbon and nitrogen and shallower thaw depth.

**Figure 4:**
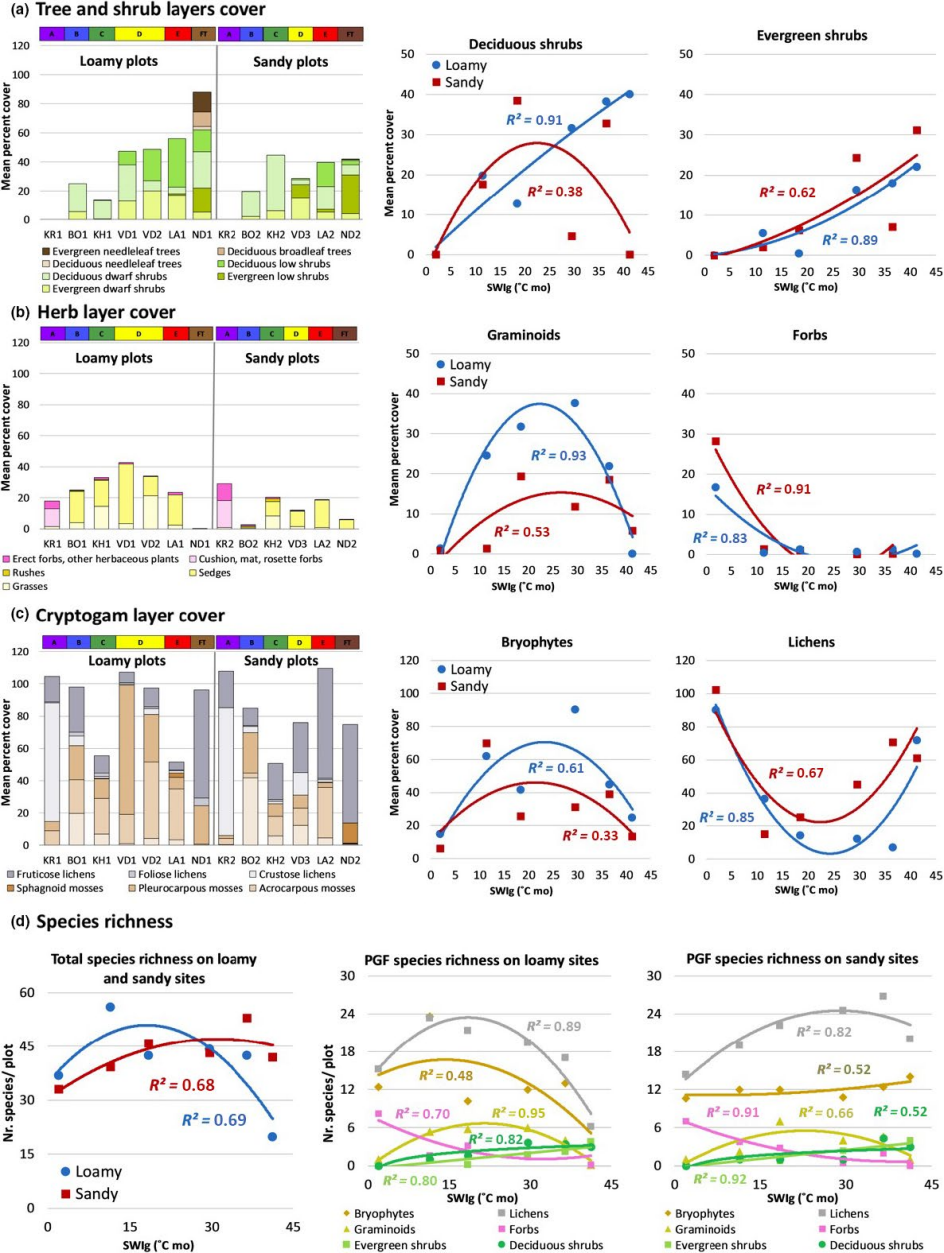
Plant‐growth‐form (PGF) cover and species richness trends along the summer‐warmth (SWI_g_) gradient. (a–c) PGF cover in the layers of the plant canopy (tree and shrub, herb and cryptogam). Left: Bar graphs of mean cover of plant growth forms at each location in loamy and sandy sites. Right: Trend lines of mean cover of major PGF groups (deciduous shrubs, evergreen shrubs, graminoids, forbs, bryophytes and lichens) vs. SWI_g_. (d) Mean species richness vs. summer warmth (SWI_g_). (a) Mean total species richness on loamy and sandy sites. (b) Mean species richness of major PFG groups on loamy sites. (c) Mean species richness of major PFG groups on sandy sites. Equations of the trend lines are in Supplementary Information, Appendix S9

The height of the plant canopy, number of canopy layers, LAI, NDVI and total phytomass all generally increase with summer warmth (Figure 4 and Supporting Information Appendix S8, Figure S8.2). The only site with trees is the Nadym forest site (ND1), which has mean total tree cover of 26% (Figure 4a, left, brown portion of stacked bars), split between evergreen needle‐leaf trees (*Pinus sylvestris* and *P. sibirica*), deciduous broad‐leaf trees (*Betula pubescens*) and deciduous needle‐leaf trees (*Larix sibirica*). (See Supporting Information Appendix S4 for the raw species cover estimates.) Low shrubs (40–200‐cm tall) occur in subzones D and E and the forest–tundra (VD1, VD2, LA1, LA2, ND1 and ND2) and are most abundant on loamy soils (Figure 4a, left). Dwarf shrubs (<40‐cm tall) occur in all subzones except subzone A, where woody plants are absent. Deciduous shrub cover (Figure 4a, centre) varies nearly linearly with SWI_g_ on loamy soils (*R* ^2^ = 0.91) and has a weak polynomial trend (*R* ^2^ = 0.38) on sandy soils. Evergreen shrub cover has an exponential trend on loamy soils (*R* ^2^ = 0.89) and sandy soils (*R* ^2^ = 0.61; Figure 4a, right). Deciduous and evergreen shrub height and LAI increase exponentially with SWI_g_ (Supporting Information Appendix S8, Figure S8‐2).

Graminoids are dominant in the herbaceous layer in all subzones except subzone A, where forbs are most abundant (Figure 4b, left). Graminoid cover peaks at 40% in subzone D on loamy soils (Figure 4b centre). On sandy soils, graminoid cover peaks at approximately 20% in subzones C and E. Sedges dominate the graminoid cover in all subzones except subzone A, where sedges are absent. Sedges have generally higher cover on loamy sites compared to sandy sites. Grass cover is highest (>14%) on loamy soils in subzones C and D. Forbs occur with low cover in all subzones except subzone A, where they are the dominant component of the vascular plant cover (Figure 4b, right).

Lichens peak at both ends of the gradient on both loamy and sandy sites (Figure 4c, left and right). Fruticose lichens have highest cover in subzone E and the forest–tundra transition, exceeding 60% cover on loamy and sandy sites in the forest–tundra transition (ND1 and ND2); whereas crustose lichens (including biological crusts) have highest cover in subzone A, exceeding 80% cover on loamy and sandy sites (KR1 and KR2). Pleurocarpous mosses (those with branching growth forms, often forming carpets) are more abundant on loamy soils; whereas acrocarpous mosses (unbranched, often smaller mosses) are more abundant on acidic soils. Bryophyte cover peaked in the central part of the SWI_g_ gradient.

The range in total species richness at seven of the 12 sites was 39–46 species/plot, with extremes of 19.8 species/plot at the FT forest loamy site and 56 species/plot at the subzone B loamy site (Supporting Information Appendix S10, Figure 4d, left). The low species richness at the FT forest site (ND‐1) is explained by the low diversity of cryptogams (6.2 lichen species and three bryophyte species), despite the very high cover of fruticose reindeer lichens. The high species richness at the subzone B loamy site (BO‐1) is partly due to the presence of patterned ground and two distinct microhabitats (non‐sorted circle centres and inter‐circle areas) within the 5 m × 5‐m plots.

The mean species richness is high in the cryptogam layer (lichens plus bryophytes, grey and brown lines in Figure 4d, centre and right), ranging between 25–47 species/plot at all sites except ND‐1, which has 9.2 species/plot. The average total species richness ranges much more narrowly between 7.8 and 13.8 in the herb and shrub layers (Figure 4d). The various PGFs reach peak mean richness at different points along the bioclimate and soil‐texture gradients: lichens, 26.8 species/plot (subzone E, sandy); bryophytes, 23.6 species/plot (subzone B, loamy); forbs, 8.2 species/plot (subzone A, loamy); graminoids, 7.4 species/plot (subzone D, loamy); deciduous shrubs, 4.4 species/plot (subzone E, sandy); evergreen shrubs, 4 specie/plot (forest–tundra transition, sandy); and trees, 3.4 species/plot (forest–tundra transition, loamy).

### 3.4 Ordination

**Figure 5:**
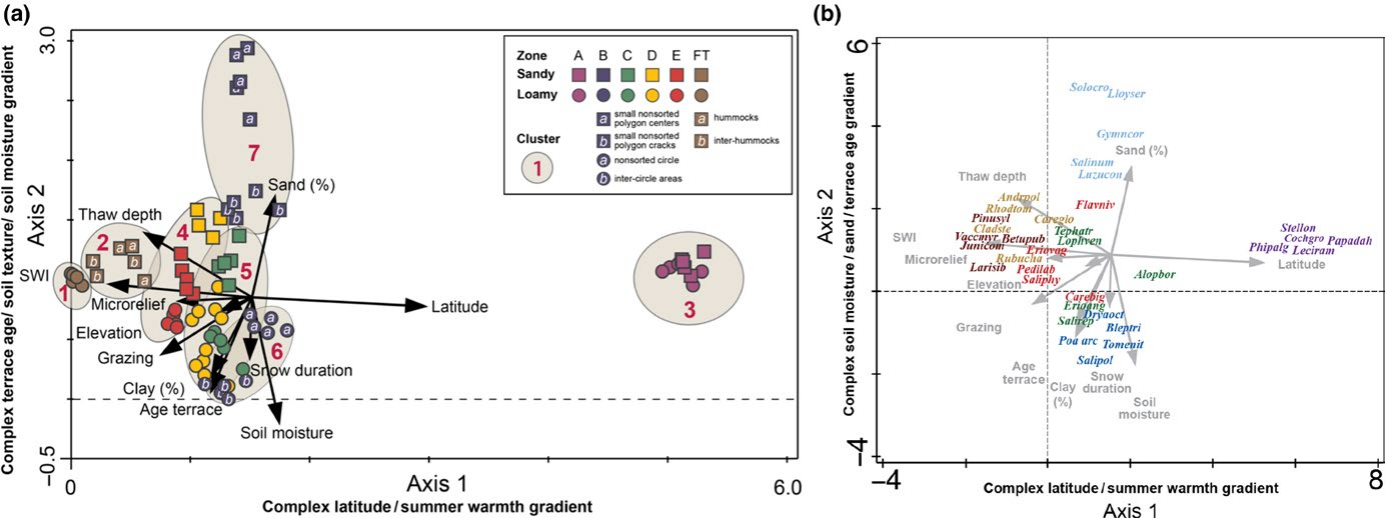
DCA ordination of EAT plots. (a) Plot ordination with environmental joint plot. Units along the axes are *SD* units, an indicator of the amount of species turnover in the data set. Four *SD* units are considered to represent approximately one complete species turnover. Plot symbols are colour‐coded according to bioclimate subzones; shapes of symbols correspond to soil texture. Small letters (a, b) are microhabitats corresponding to patterned ground features at the Nadym Site ND‐2 (hummocks and inter‐hummocks) and Ostrov Belyy Site OB‐1 (non‐sorted circles and inter‐circle areas) and Site OB‐2 (small non‐sorted polygon centres and cracks). Red cluster numbers are according to clusters in Figure 3. Joint‐plot arrows denote direction and strength of correlations with environmental variables with *p* ≤ 0.05. (b) Species ordination. Centres of distributions are shown for the top five diagnostic taxa in each cluster. The alphabetic taxon codes are abbreviations containing the first four letters of the genus and first three letters of species names. Colours of taxa labels correspond to dominant bioclimate subzones of the clusters for which the taxa are diagnostic (Dark brown, cluster 1, FT‐Forest; light brown, cluster 2, FT‐tundra; red, cluster 4, subzone E & subzone D, sandy; green, cluster 5, subzone D, loamy & subzone C; dark blue, cluster 6, subzone B, loamy; light blue, cluster 7, subzone B, sandy; purple, cluster 3, subzone A.

The DCA plot ordination (Figure 5a) displays the 76 plots according to their respective bioclimate subzone, texture class and cluster. Axis 1 has a high positive linear correlation with latitude (0.96) and a high negative correlation with SWIg (−0.77) (Supplementary Information Appendix S11). Plots in subzone A (cluster 3) are geographically and floristically widely separated from plots in the rest of the clusters, which form a large megacluster toward the left side of the ordination. Within the megacluster, there is generally a clear separation of plots in each of the statistical clusters, with transition from the relatively warm FT sites (clusters 1 and 2) on the left side of the megacluster to relatively cold subzone B (clusters 6 and 7) on the right side. There is relatively high floristic similarity among most of the plots in this megacluster, particularly among clusters 4, 5 and 6, indicating a continuous floristic gradient along the main Yamal Peninsula, rather than distinct vegetation units in each bioclimate subzone. Axis 2 has a strong positive correlation with sand percentage (0.64) and a strong negative correlation with soil moisture and terrace age (−0.75 and 0.51, respectively) (Supplementary Information Appendix S11). All sandy sites (coloured squares) are in the upper part the ordination, and loamy sites (coloured circles) are in the lower part.

The species ordination (Figure 5b) displays the centroids of distribution of five taxa with the highest fidelity to each of the seven clusters (35 total taxa). As expected, the centres of distributions for the diagnostic taxa generally align with the areas of the clusters for which they are diagnostic.

## 4 DISCUSSION AND CONCLUSIONS

### 4.1 Mesic vegetation transitions along the EAT summer temperature gradient

A primary motivation for this study was to develop a baseline of ground‐based vegetation information along the complete Arctic summer temperature gradient in the maritime Arctic portion of western Russia to support remote sensing interpretations. We sampled and analysed plant communities on homogeneous mesic sites with loamy and sandy soils along the summer temperature gradient of the EAT. Satellite‐derived summer land‐surface temperatures (Comiso, 2006; Raynolds, Comiso, Walker, & Verbyla, 2008) provided a consistent spatial record of mean summer ground‐surface temperatures (SWI_g_) across the full length of the EAT, including locations where station data were unavailable.

The EAT analysis focused on mesic tundra areas where climate is the primary factor controlling the character of the vegetation. Although we initially considered these mesic sites to be zonal habitats, it soon became clear that the tundra over nearly the entire Yamal Peninsula is strongly influenced by a long history of reindeer grazing. The only locations that were free of recent reindeer foraging were Krenkel and Nadym at the extreme northern and southern ends of the bioclimate gradient. Both of these sites had high cover of lichens, indicating that reindeer at the other sites have greatly reduced the lichen cover. Reindeer herds graze heavily on lichens particularly during the snow‐covered months of winter and spring. The results of our study and others (Pajunen, 2009; Pajunen, Virtanen, & Roininen, 2008; Vowles, Lovehav, Molau, & Björk, 2017; Yu, Epstein, Walker, Frost, & Forbes, 2011) and comparison with results from a similar transect in North America where there are relatively low *Rangifer* densities (Walker, Epstein, et al., 2012) indicate that the reindeer have had a long‐term major impact on the shrub, graminoid and moss layers on the Yamal (Forbes et al., 2009). Quantifying this effect is difficult because of lack of reindeer exclusion areas.

Vegetation units described here for the middle portion of the EAT bioclimate gradient display gradual floristic transitions between bioclimate subzones and are only weakly aligned with previously described Br.‐Bl. classes. A formal association‐level classification for the Yamal region should await a broader analysis that includes new data collected within the past few years. Data from both the EAT and NAAT transects and additional data from zonal sites elsewhere in the Arctic should be used to develop a unified Braun‐Blanquet classification for zonal vegetation across the full Arctic bioclimate gradient using the habitat‐based approach of Mucina et al., 2016; Walker et al., 2018). There is especially a need for a new Br.‐Bl. class corresponding to zonal acidic tundra in the middle part of Arctic bioclimate gradient. Additional studies are needed to develop clear Br.‐Bl. syntaxa to characterize the variation along other important habitats and environmental gradients across the Arctic, including representative toposequences, riparian chronosequences, snowbed gradients and major disturbance gradients.

The analyses of trends of PGF cover and species richness within canopy layers vs. mean SWI_g_ provided quantitative data across the bioclimate gradient that support the observations of other investigators including: (a) the occurrence of progressively more and taller layers in the plant canopy with warmer temperatures (Elmendorf et al., 2012; Matveyeva, 1998), (b) increases in vascular plant cover and diversity along the summer temperature gradient (Daniëls et al., 2013; Rannie, 1986; Young, 1971), and (c) exclusion of woody plants, sedges and *Sphagnum* peat from the northernmost subzone A (Yurtsev, 1994b). While cover and species richness of evergreen and deciduous shrubs generally increased with higher SWI_g_, cover of lichens and forbs declined. Graminoid cover and species richness of lichen and bryophyte species richness showed parabolic trends with maximum values in the central part of the temperature gradient.

Much recent research regarding productivity patterns in the Arctic has focused on the increased abundance of shrubs associated with warming temperatures, which are thought to be a primary cause of the recent increases in NDVI observed in satellite data (Myers‐Smith et al., 2011). Our study documented strong, mostly positive, exponential trends with SWI_g_ for deciduous and evergreen shrub cover, shrub layer height, herb layer height, litter cover, LAI, NDVI and above‐ground phytomass. The study also documented the dominance of shrubs in the Low Arctic (subzones E and D), dwarf shrubs, graminoids and bryophytes in the Middle Arctic (subzones C and B), and forbs and crustose lichens in the extreme High Arctic.

### 4.2 The role of soil texture

The floristic contrast between the loamy and sandy sites varies considerably between locations across the EAT, a result of much greater site‐factor heterogeneity of the sandy sites. The Nadym and Ostrov Belyy locations illustrate rather extreme contrasts in ecosystem structure that can occur on loamy vs. sandy soils. At Nadym, the site on the sandy, relatively young surface at ND‐1 is relatively well drained, has no permafrost and is forested; whereas the ND‐2 site on older, more fine‐grained soils is ice‐rich, relatively poorly drained, and covered with hummocky tundra vegetation (Supporting Information Appendix S3, Figure S3‐6). A host of site factors interact to affect the vegetation structure and composition at this site, including much thicker soil organic layers, thin active layers, relatively cold soils and very low CECs on the older loamy soils. A similar contrast occurred at Ostrov Belly (Supporting Information Appendix S3, Figure S3‐2) and is illustrated in the numerical classification and DCA ordination, where the sandy and loamy plots are placed in separate clusters (Figure 3, clusters 6 and 7) and are widely separated along Axis 2 of the ordination (Figures 3 and 5). The sandy sites at Ostrov Belyy are much drier than the loamy sites at this location and have many other site factor differences that separate them.

The opposite situation occurs at Krenkel (subzone A; Supporting Information Appendix S3, Figure S3‐1), where both study sites have similar site factors with high floristic similarity and are placed in a single tight cluster in the ordination (cluster 3 in Figures 3 and 5). Loamy and sandy sites at Laborovaya (subzone E; Supporting Information Appendix S3, Figure S3‐5) also have high floristic similarity, but in this case, there is also relatively high similarity with the sandy sites at Vaskiny Dachi (Supporting Information Appendix S3, Figure S3‐4), so all three sites (LA‐1, LA‐2, VD‐2,) are placed in a single numerical cluster (cluster 4 in Figures 3 and 5), with several acidophilic, oligotrophic, hypoarctic diagnostic species.

Part of the explanation for much larger variation in the sandy sites is that during site selection, it was relatively easy to find large sites to sample vegetation on mesic silt loam to sandy loam soils, whereas the availability of mesic very sandy sites was more limited. The relatively young sandy sites are also more susceptible to disturbance by reindeer and strong winds, whereas the older loamy sites have tended to stabilize toward the regional zonal conditions.

### 4.3 Special importance of subzone A

A major accomplishment of this study was the first detailed vegetation description from exceptionally cold, wet and windy Hayes Island. Our results documented the high floristic dissimilarity of Hayes Island to the rest of the EAT (Figure 5), the dominance of biological soil crusts in the cryptogam layer and the dominance of forbs among the vascular plants (Figure 4b). It revealed a vegetation composed mainly of biological soil crusts, where even the vascular plants in the herb layer have cryptogam‐like cushion and mat growth forms, unlike any other site along the EAT. Sites not exposed to excessive wind erosion had unexpectedly high hand‐held NDVI (0.44–0.48), most likely caused by the high cover of wet biological soil crusts, which covered 50%–85% of the soil surface and comprised 33%–86% of the total biomass (Walker, Epstein, et al., 2012; Walker, Frost, et al., 2012). Rich fruticose lichen communities occurred on the most favourable zonal sites on Hayes Island, a result of the absence of reindeer (Supporting Information Appendix S12).

Numerous other studies have also noted the unique vegetation in subzone A (Chernov & Matveyeva, 1997; Daniëls et al., 2016) and its extreme susceptibility to climate change (Walker, Raynolds, & Gould, 2008). It is interesting that the total species richness of the coldest, most northern zonal location (Krenkel, KR‐1, 37 species) is higher than that of the warmest most southern zonal location (Nadym, ND‐1, 20 species; Supporting Information Appendix S12). The relatively high species richness at Krenkel is due to the large number of cryptogam species (24–27.8 species). Other arctic researchers have also noted high plot‐scale cryptogam species richness at cold temperatures (Bültmann, 2005; Lünterbusch & Daniëls, 2004; Matveyeva,^1998^; Timling et al., 2012). In studies of Arctic lichen floras from subzone E to subzone A, the number of vascular plant species declines by approximately 95%, whereas the number of lichen species declines by only approximately 15% (Dahlberg, Bültmann, & Meltofte, 2013). The same authors note that the relatively small decline in lichen species at higher latitudes is due mainly to reductions in the number of lichens that normally grow on woody plants, which are greatly reduced toward the north. Increased availability of light due to reduced competition from herbs and shrubs is a major cause of high moss and lichen richness at the more northern sites (Marshall & Baltzer, 2015; Walker et al., 2006). Further competition for light occurs within very dense cryptogam layers in the southern locations, where a few reindeer lichen species with erect fruticose lichen growth forms (e.g. *Cladonia stellaris*,* C. stygia, C. rangiferina*,* C. arbuscular* and *C. mitis*) densely cover the ground of lichen woodlands and out‐compete other species.

### 4.4 Implications for Arctic climate change and ecosystem studies

Ground‐based documentation of existing patterns of vegetation is a critical element of space‐based monitoring of changes to terrestrial ecosystems during a time of rapid climate and land‐use change in the Arctic (Stow et al., 2004). The patterns of vegetation greenness (NDVI) change have not been spatially or temporally consistent across the Arctic, due in part to the constantly changing patterns of sea ice in the Arctic basin (Bhatt et al., 2013) and changes in the growing season and productivity patterns ((Park et al., 2016). Although difficult logistics limit the number of sampling locations and the quantity of data that can be collected in the vast landscapes of the Arctic, there were advantages of these constraints during our studies because they facilitated interdisciplinary teamwork at the selected sites, assuring a largely spatially coherent database of vegetation, soil, permafrost and remote‐sensing information to aid remote sensing interpretations and vegetation change modelling along a full maritime Arctic climate gradient. The research sites are permanently marked and provide a baseline against which to measure future vegetation change. The data should prove useful for interpretations of change to a wide variety of ecosystem properties and functions, including shrub growth (Myers‐Smith et al., 2011), permafrost regimes (Romanovsky et al., 2017), Arctic tree lines (Harsch, Hulme, McGlone, & Duncan, 2009), snow distribution (Brown et al., 2017), regional hydrology (Prowse et al., 2017), soil carbon fluxes (Christensen et al., 2017), biodiversity (Meltofte, 2013) and land‐use changes (AMAP 2010; Nymand & Fondahl, 2014). As sea ice retreats, it will be important to continue monitoring the changes from space, and also to continue to obtain ground‐based information to document the consequences for the land surface (Bhatt et al., 2014). This is especially important in subzone A, which should be considered an endangered bioclimate subzone (Walker, Raynolds, et al., 2008).

## Supporting information


**Appendix S1**. Geological setting of the Yamal Peninsula.
**Appendix S2**. Typical plot layout.
**Appendix S3**. Eurasia Arctic Transect location and site descriptions.
**Appendix S4**. Eurasia Arctic Transect species cover‐abundance data.
**Appendix S5**. Eurasia Arctic Transect environmental data.
**Appendix S6**. Full synoptic table.
**Appendix S7**. Diagnostic, constant, and dominant taxa for EAT clusters.
**Appendix S8**. Trends of selected soil and vegetation properties vs. summer warmth index.
**Appendix S9**. Regression equations for trend lines of analysed variables.
**Appendix S10**. Number of species per plot along the Eurasia Arctic Transect.
**Appendix S11**. Correlations between four axes of the DCA ordination and environmental variables.
**Appendix S12**. Lichen‐rich tundra of Hayes Island.
