# Supplementary material for: Vegetation on mesic loamy and sandy soils along a 1700‐km maritime Eurasia Arctic Transect
Source: Appl Veg Sci. 2019 Feb 27;22(1):150–67. doi: 10.1111/avsc.12401 (PMC6519894; doi:10.1111/avsc.12401)
Supplement: Supplementary file 1 — Appendix S1. Geological setting of the Yamal Peninsula. Appendix S2. Typical plot layout. Appendix S3. Eurasia Arctic Transect location and site descriptions. Appendix S4. Eurasia Arctic Transect species cover‐abundance data. Appendix S5. Eurasia Arctic Transect environmental data. Appendix S6. Full synoptic table. Appendix S7. Diagnostic, constant, and dominant taxa for EAT clusters. Appendix S8. Trends of selected soil and vegetation properties vs. summer warmth index. Appendix S9. Regression equations for trend lines of analysed variables. Appendix S10. Number of species per plot along the Eurasia Arctic Transect. Appendix S11. Correlations between four axes of the DCA ordination and environmental variables. Appendix S12. Lichen‐rich tundra of Hayes Island. [file AVSC-22-150-s001.zip › supinfo/Appendix_S11_CorrelationsDCAaxesXEnvir_variables_20190210.pdf]

Supporting information, Appendix S11. Intraset correlation coefficients between four axes of the DCA ordination and tested environmental variables. Coefficients are species-environment correlations, which measure the strength of the relationship between species or environmental variables and the ordination axes in the program CONOCO, which was used for the DCA analysis via JUICE.

**Expl Ax1**	1													
**Expl Ax2**	0.1549	1												
**Expl Ax3**	0.0006	-0.0507	1											
**Expl Ax4**	0.0847	-0.2418	0.2178	1										
**ELEV**	-0.1735	-0.1239	-0.0163	-0.2228	1									
**MICRORLF**	-0.4085	-0.0986	0.2855	-0.1293	0.4592	1								
**THAW_DEP**	-0.572	0.3622	-0.0812	-0.4036	0.2749	0.6013	1							
**SOIL_MOI**	0.1054	-0.8775	-0.1302	0.044	0.1771	-0.1041	-0.539	1						
**SNOW_DUR**	-0.0342	-0.4383	0.7361	0.043	-0.0098	0.2526	-0.111	0.2148	1					
**SWI**	-0.7742	-0.0403	0.1425	0.0133	0.5721	0.5997	0.728	-0.2509	0.1039	1				
**SAND(%)**	0.1638	0.7489	0.021	-0.3403	-0.1275	-0.2667	0.3302	-0.6097	-0.4072	-0.0512	1			
**CLAY(%)**	-0.1895	-0.4958	0.3005	0.128	0.1423	0.3854	-0.2195	0.3829	0.5956	0.1006	-0.8051	1		
**Latitude**	0.9595	0.1022	-0.0296	0.1314	-0.4014	-0.5264	-0.6893	0.1488	-0.0386	-0.9085	0.1032	-0.1611	1	
**AgeTerac**	-0.2494	-0.5939	-0.4808	0.271	0.2526	0.1143	-0.0656	0.4106	-0.0596	0.2563	-0.6914	0.318	-0.2414	1
	**Expl Ax1**	**Expl Ax2**	**Expl Ax3**	**Expl Ax4**	**ELEV**	**MICRORLF**	**THAW_DEP**	**SOIL_MOI**	**SNOW_DUR**	**SWI**	**SAND(%)**	**CLAY(%)**	**Latitude**	**AgeTerac**
