# Supplementary material for: Vegetation on mesic loamy and sandy soils along a 1700‐km maritime Eurasia Arctic Transect
Source: Appl Veg Sci. 2019 Feb 27;22(1):150–67. doi: 10.1111/avsc.12401 (PMC6519894; doi:10.1111/avsc.12401)
Supplement: Supplementary file 1 — Appendix S1. Geological setting of the Yamal Peninsula. Appendix S2. Typical plot layout. Appendix S3. Eurasia Arctic Transect location and site descriptions. Appendix S4. Eurasia Arctic Transect species cover‐abundance data. Appendix S5. Eurasia Arctic Transect environmental data. Appendix S6. Full synoptic table. Appendix S7. Diagnostic, constant, and dominant taxa for EAT clusters. Appendix S8. Trends of selected soil and vegetation properties vs. summer warmth index. Appendix S9. Regression equations for trend lines of analysed variables. Appendix S10. Number of species per plot along the Eurasia Arctic Transect. Appendix S11. Correlations between four axes of the DCA ordination and environmental variables. Appendix S12. Lichen‐rich tundra of Hayes Island. [file AVSC-22-150-s001.zip › supinfo/Appendix_S12_Photos_of_lichen-rich_tundra,_Krenkel_20190210.pdf]

Supporting Information, Appendix S12. Photos of lichen-rich tundra of Hayes Island.

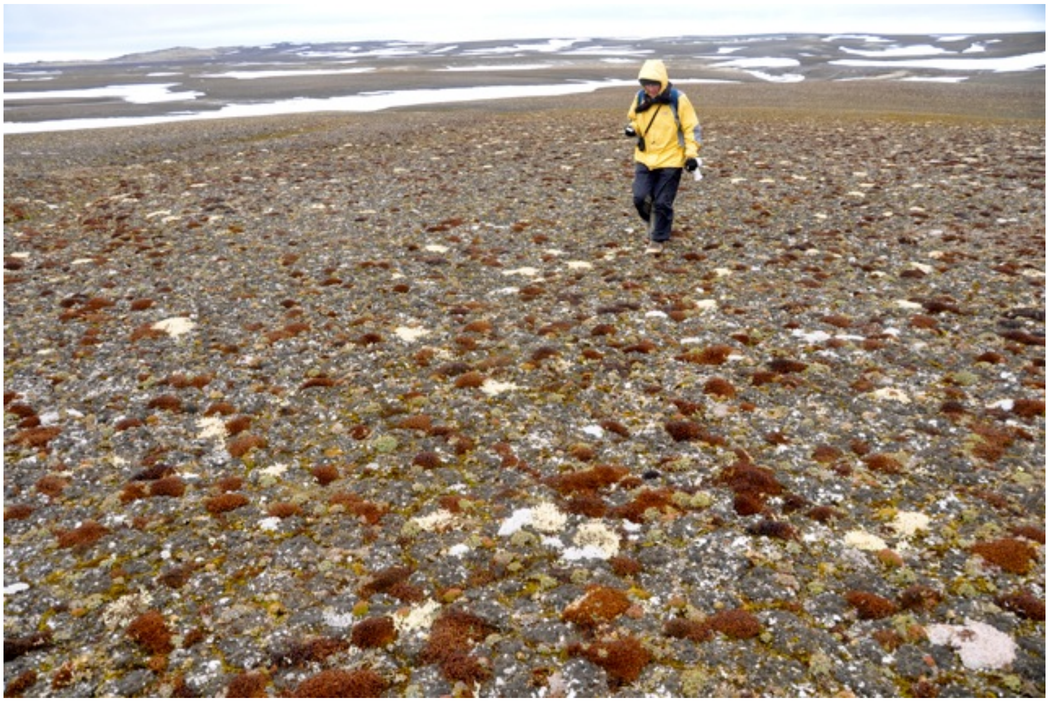

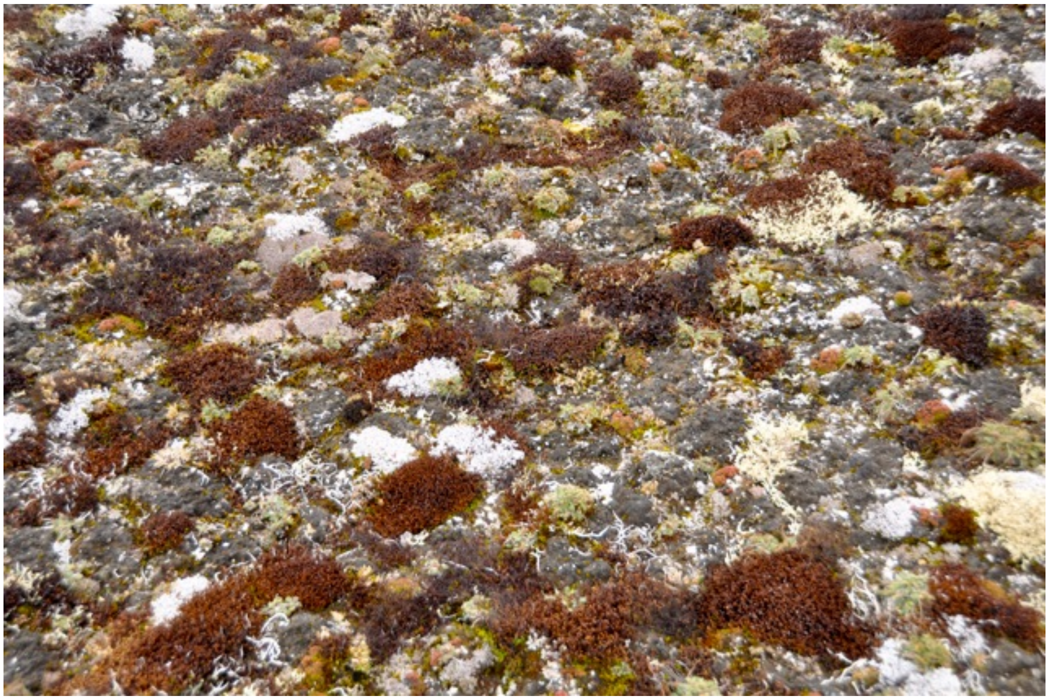

Lichen-rich tundra of Hayes Island. The rich lichen cover is able to develop in the cold wet arctic maritime climate and thrives because of the lack of competition from other growth forms and the lack of reindeer on the island. The brown lichens are mainly *Cetrariella delisei* and *Cetraria islandica*. The dominant white lichens are *Stereocaulon alpinum* and *Thamnolia subuliformis*. The yellowish lichens are mainly *Flavocetraria cucullata*. These communities were discovered on the last day of the 2010 expedition and unfortunately were not sampled. Photos: D.A. Walker
