# Supplementary material for: Vegetation on mesic loamy and sandy soils along a 1700‐km maritime Eurasia Arctic Transect
Source: Appl Veg Sci. 2019 Feb 27;22(1):150–67. doi: 10.1111/avsc.12401 (PMC6519894; doi:10.1111/avsc.12401)
Supplement: Supplementary file 1 — Appendix S1. Geological setting of the Yamal Peninsula. Appendix S2. Typical plot layout. Appendix S3. Eurasia Arctic Transect location and site descriptions. Appendix S4. Eurasia Arctic Transect species cover‐abundance data. Appendix S5. Eurasia Arctic Transect environmental data. Appendix S6. Full synoptic table. Appendix S7. Diagnostic, constant, and dominant taxa for EAT clusters. Appendix S8. Trends of selected soil and vegetation properties vs. summer warmth index. Appendix S9. Regression equations for trend lines of analysed variables. Appendix S10. Number of species per plot along the Eurasia Arctic Transect. Appendix S11. Correlations between four axes of the DCA ordination and environmental variables. Appendix S12. Lichen‐rich tundra of Hayes Island. [file AVSC-22-150-s001.zip › supinfo/Appendix_S1_Geological_setting_of_the_Yamal_Peninsula_20190210.pdf]

Supporting Information, Appendix S1.
Marine and alluvial terraces of the Yamal Peninsula in relation to soil texture

Marine and alluvial terraces of varying age occur at all the locations along the Eurasia Arctic Transect (EAT) (Fig. S1-1). These terraces were formed during the postglacial emergence of northern Eurasia and the Franz Josef Land archipelago (Dibner, 1965; Forman et al., 2004; Ingólfsson, Möller, & Lokrantz, 2008; Saks, 1953; Svendsen et al., 2004). Relevant to this study, the older terraces (terraces III to V) generally have finer-grained loamy soils, and the younger terraces have sandy soils, providing the opportunity to compare differences in vegetation with respect to soil texture along the full bioclimate gradient. The older terraces also often have high concentrations of massive ground ice, and are extensively eroded by thawing permafrost and landslides (Ukraintseva, 2008, 2010). We selected loamy sites on broad well-drained hilltops of older terraces (III and IV) and sandy sites on younger terraces (I and II). (See Table 1 of main paper).

References:

Dibner, V. D. (1965). The history of late Pleistocene and Holocene sedimentation in Franz Josef Land (in Russian). *Transactions of the Scientific Research Institute of the Geology of the Arctic*, *143*, 300–318.

Forman, S. L., Lubinski, D. J., Zeeberg, J. J., Snyder, J. A., Siegert, M. J., & Matishov, G. G. (2004). A review of postglacial emergence on Svalbard, Franz Josef Land and Novaya Zemlya, northern Eurasia.

Ingólfsson, Ó., Möller, P., & Lokrantz, H. (2008). Late Quaternary marine-based Kara Sea ice sheets: a review of terrestrial stratigraphic data highlighting their formation. *Polar Research*, *27*(2), 152–161. http://doi.org/10.3402/polar.v27i2.6173

Saks, V. N. (1953). Quaternary period in the Soviet Arctic (in Russian) (pp. 1–627). Leningrad: Vdtransizdat.

Svendsen, J. I., Alexanderson, H., Astakhov, V., Demidov, I., Dowdeswell, J. A., Funder, S., et al. (2004). Late Quaternary ice sheet history of northern Eurasia. *Quaternary Science Reviews*, *23*(11-13), 1229–1271. http://doi.org/10.1016/j.quascirev.2003.12.008

Ukraintseva, N. (2008). Vegetation response to landslide spreading and climate change in the West SiberianTundra. In *Ninth International Conference on Permafrost* (pp. 1793–1798). Fairbanks.

Ukraintseva, N. (2010). High willow shrubs in Yamal: reasons of their wide expansion and methods of biomass assessment. Presented at the Second Yamal Land-Cover Land-Use Change Workshop, Rovaniemi, Finland.

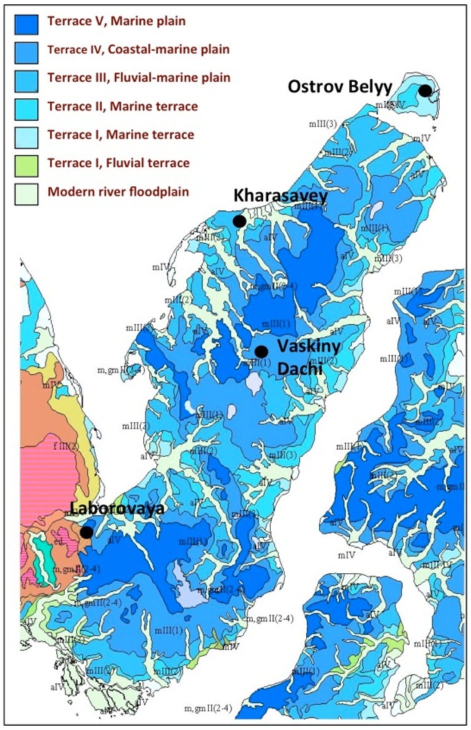

Figure S1-1. Quaternary-age terraces of the Yamal Peninsula. Terrace ages are still disputed; dates provided here are based mainly on Svendsen et al. (2004), with local glacial age names provided by the Earth Cryosphere Institute. Vegetation on starred (*) terraces was sampled during the EAT studies. *Terrace I, 7-12 m a.s.l., Sartansky-age (Last Glacial Maximum, Late Weichselian), ≈ 10-25 ka; *Terrace II, 10-25 m a.s.l., Karginsky-Zyransky-age (Middle Weichselian), ≈ 25-75 ka; *Terrace III, 26-40 m a.s.l., Ermanovsky-age (Early Weichselian), ≈ 75-117 ka; *Terrace IV, 40-45 m a.s.l., Kazantsevskaya-age (Eemian interglacial), ≈ 117-130 ka; Terrace V, 45-58 m a.s.l., Salekhardskaya age (Saalian), ≈130- 200 ka. The younger terraces (I, II) generally have sandy soils. Not shown are the marine terraces of Hayes Island (Dibner 1965). Sites on Hayes Island, FJL, are on sandy marine terraces at approximately 30 m a.s.l. and 10 m a.s.l. Graphic: Earth Cryosphere Institute.
