# Supplementary material for: Vegetation on mesic loamy and sandy soils along a 1700‐km maritime Eurasia Arctic Transect
Source: Appl Veg Sci. 2019 Feb 27;22(1):150–67. doi: 10.1111/avsc.12401 (PMC6519894; doi:10.1111/avsc.12401)
Supplement: Supplementary file 1 — Appendix S1. Geological setting of the Yamal Peninsula. Appendix S2. Typical plot layout. Appendix S3. Eurasia Arctic Transect location and site descriptions. Appendix S4. Eurasia Arctic Transect species cover‐abundance data. Appendix S5. Eurasia Arctic Transect environmental data. Appendix S6. Full synoptic table. Appendix S7. Diagnostic, constant, and dominant taxa for EAT clusters. Appendix S8. Trends of selected soil and vegetation properties vs. summer warmth index. Appendix S9. Regression equations for trend lines of analysed variables. Appendix S10. Number of species per plot along the Eurasia Arctic Transect. Appendix S11. Correlations between four axes of the DCA ordination and environmental variables. Appendix S12. Lichen‐rich tundra of Hayes Island. [file AVSC-22-150-s001.zip › supinfo/Appendix_S3_EAT_site_descriptions_20190210.pdf]

Supporting Information, Appendix S3.
Eurasia Arctic Transect location and site descriptions

This appendix contains brief descriptions of research locations and study sites along the Eurasia Arctic Transect (EAT). Table 1 in the main paper provides a summary of locations, site numbers, site names, microsites, geological settings, marine and alluvial terraces, parent material, and dominant vegetation. Table 2 in the main paper provides the summary of climate information. More complete descriptions with additional photographs are in the project data reports (Walker et al., 2011; 2008; Walker, Epstein, et al., 2009a; Walker, Orekhov, et al., 2009b).

Krenkel

a. **b.**

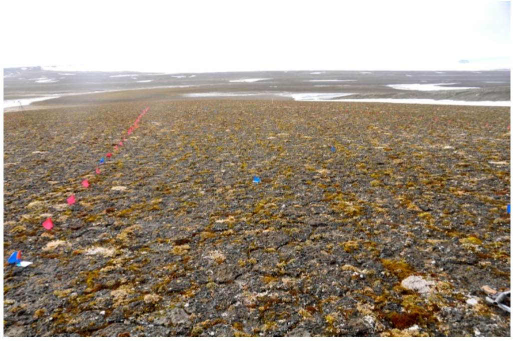 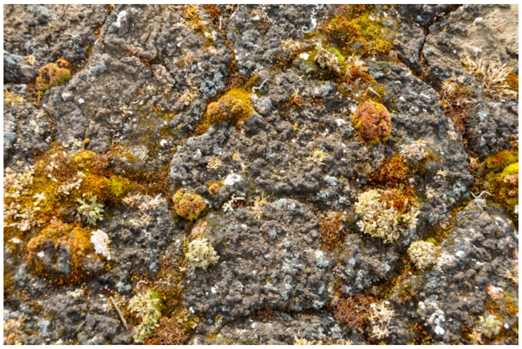

c. **d.**

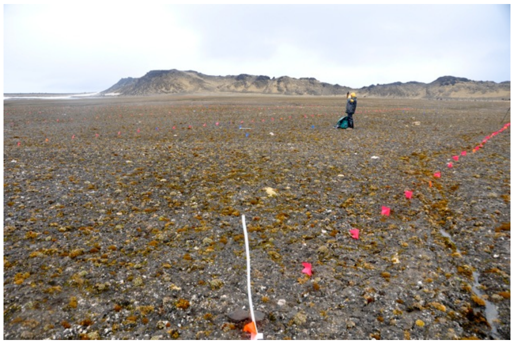 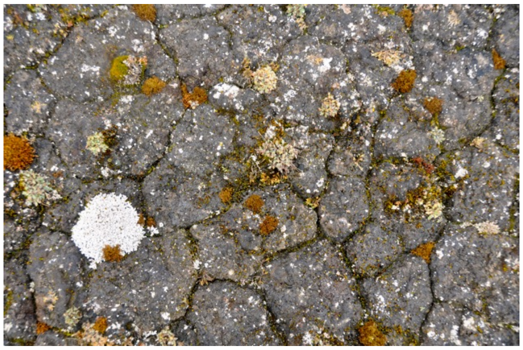

Figure S3-1. Vegetation of the Krenkel (KR) EAT study location. a. KR, sandy-loam site, landscape view**. b.** Close-up of vegetation of KR-1. Dominant vascular plant species are *Papaver dahlianum* spp*. polare, Stellaria edwardsii, S. crassipes, Draba micropetala, Saxifraga cespitosa,* and *Phippsia algida*. Cushion forms of lichens and mosses include *Cetrariella delesii, C. islandica, Flavocetraria cucullata, Thamnolia subuliformis, Stereocaulon alpinum and S. rivulorum, Polytrichastrum alpinum, Orthothecium chryseon,* and *Bryum rutilans*. **c.** KR-2, sandy site, landscape view**. d.** Close up of the vegetation of KR-2. The dominant vascular plants are *Papaver dahlianum* spp*. polare, Stellaria edwardsii, S. crassipes, Saxifraga cernua, Phippsia algida* and *Cochlearia groendlandica.* Important cryptogamic species include the white lichen *Stereocaulon alpinum*. Note the small nonsorted polygons with plants growing preferentially in the cracks between polygons; 50-85% of the polygon surfaces are covered by gray biological soil crusts, which include *Anthelia juratzkana, Protopannaria pezizoides, Lecidea ramulosa, Baeomyces rufus*, *Lepraria gelida, Ochrolechia inaequatula*, *Ochrolechia frigida*, *Pertusaria* cf. *coriacea,* unidentified lichen prothalli, and algal crusts. Photos: D.A. Walker.

Hayes Island is a small 132-km^2 landmass in the central part of the Franz Jozef Land (FJL) archipelago. Unlike most islands in FJL, the majority of Hayes Island is ice free, with exception of the small (approximately 20 km^2) semi-circular-shaped Hydrogeographers Ice Cap on the northern coast of the island. Our studies were conducted near the Krenkel Hydrometeorological Station in the northeast corner of the island (80˚ 37’ N, 58˚ 03’ E).

The lithostratigraphy and geomorphology of the archipelago are more similar to those of Svalbard and Sverdrup basin in Canada than they are to the Yamal Peninsula. Troughs of rift origin separate the islands and are overlain by thick sedimentary sequences (Dibner, 1965). Basalt cliffs occur along the southern coast of Hayes Island, and numerous volcanic dikes cross the island to provide

varied topography with rugged ridges and pinnacles. Most of the island is hilly and covered with sandy sedimentary deposits that are eroded by snow-melt streams. Mesozoic-age sandstones outcrops occur along stream channels, hills, and near Hydrographers Ice Cap, forming badland topography in some areas (Koryakin & Shipilov, 2009). Along the coast, unconsolidated marine deposits up to 10 m thick were formed as the island emerged following the last glacial maximum (Lubinski et al. 1999, Forman et al. 2004).

The maritime influence of the Barents Sea has a strong cooling effect on summer temperatures of the island. Cloudiness, summer fog and frequent storms are typical. High relative air humidity (80-92%) occurs all the year. The mean annual precipitation is 282 mm with the maximum precipitation occurring during October to March. The mean July temperature is only 1 ˚C, and the air summer warmth index (SWI_a) is a remarkably low 1.1 ˚C mo. The satellite-derived ground SWI_g is 1.86 ˚C mo. Strong northeasterly to southeasterly winds predominate in the winter, spring, and fall forming deep snow drifts that persist all summer in the stream networks. Hurricanes with the wind speeds up to 40 m/s are possible during this period. Extreme winds are comparatively rare in the summer.

Hayes Island has the most unique vegetation along the EAT. It is located in the Polar Desert geobotanical subregion (Alexandrova, 1980) and bioclimate subzone A of the Circumpolar Arctic Vegetation Map (CAVM Team 2003). No previous vegetation surveys are known from the island. Two sites were selected for the vegetation surveys.

KR-1, loamy site (Fig. S3-1a and b), is located on a gentle west-facing slope at an elevation of 30 m with relatively abundant plant cover. The surficial deposits are deluvium derived from the unconsolidated sandstone bedrock. Small non-sorted polygons, 10–15 cm in diameter, are common on most surfaces. These are formed by seasonal frost cracking. The cracks between the small polygons are protected habitats for small mosses, lichens and forbs (Fig. S3-1b). We did not separate the microhabitats associated with centers and cracks of these polygons, as we did on Ostrov Belyy, because of the small size of the polygons and difficulty in defining the boundaries of the communities.

KR-2, sandy site (Fig. S3-1c and d), is located on a flat sandy marine terrace at about 10-m elevation. The site has scattered glacially derived rocks. The surface has large flat-centered ice-wedge polygons 20–25 m in diameter, within these are small nonsorted polygons 10–20 cm in diameter. Differences between the two sites are rather small compared to the other EAT locations. KR-2 is more sparsely vegetated than KR-1 with about 7–15% cover of vascular plants. Cryptogamic crusts are more abundant than at KR-1, covering about 80–85% of the surface. The dominant vascular plants at both sites are *Papaver dahlianum* spp*. polare, Stellaria edwardsii, S. crassipes, Saxifraga cernua, Phippsia algida* and *Cochlearia groendlandica.* Cushion forms of the lichens *Cetrariella delesii, C. islandica, Flavocetraria cucullata, Thamnolia subuliformis, Stereocaulon alpinum and S. rivulorum* are common. Common bryophytes include *Polytrichastrum alpinum, Orthothecium chryseon, Bryum rutilans* and *Anthelia juratzkana.*

Ostrov Belyy (White Island)

a. **b.**

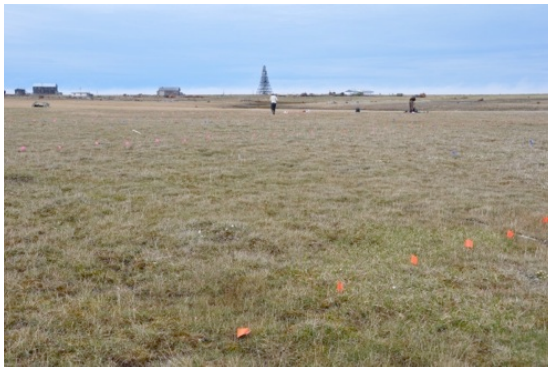

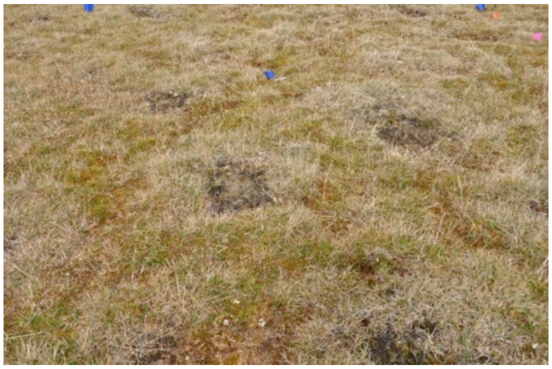

c. **d.**

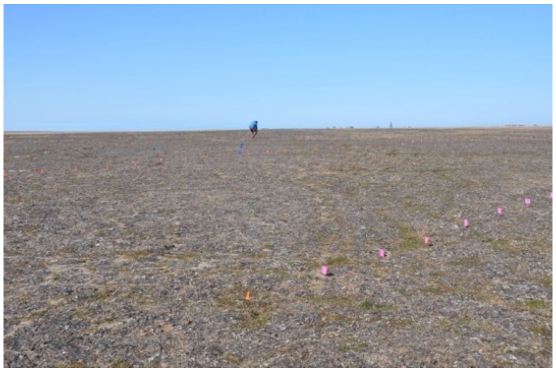

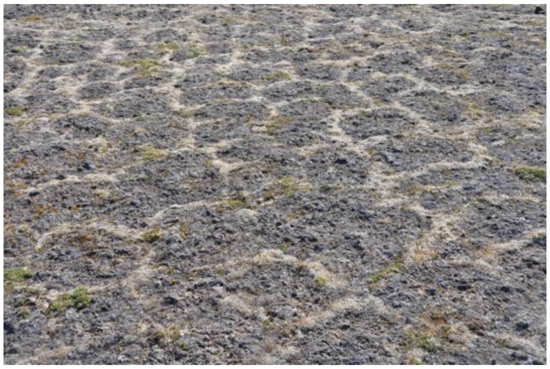

Figure S3-2. Vegetation of the Ostrov Belyy (BO) EAT study location. a. BO-1, loamy site, landscape view from the southeast corner. The polar station is in the background. **b.** Vegetation and nonsorted circles within OB-1. Diameter of the circles is approximately 50 cm. The common plant species in the moist graminoid-dominated areas between the circles include *Carex bigelowii, Salix polaris, Calamagrostis holmii, Arctagrostis latifolia, Poa arctica, Hylocomium splendens, Aulacomnium turgidum, Dicranum spp., Ptilidium ciliare, Polytrichum strictum, Sphaerophorus globosus,* and *Cladonia arbuscula.* The centers of circles are more barren and drier; the dominant species are *Dryas integrifolia, Arctagrostis latifolia, Salix polaris, Racomitrium lanuginosum, Sphaerophorus globosus, Ochrolechia frigida, Bryocaulon divergens* and *Anthelia juratskana*). **c.** BO-2, sandy site, landscape view. **d.** Vegetation and small nonsorted polygons at OB-2. Diameter of the polygons is about 20-50 cm. The gray crust is composed primarily of the liverwort, *Gymnomitrion corallioides*. The moss in the polygon cracks is *Racomitrium lanuginosum*. Other common species include *Salix nummularia* and *Luzula confusa*. (Photos: D.A. Walker.)

Ostrov Belyy is just north of the Yamal Peninsula in the Kara Sea. The 15–30-km wide Malygin Strait separates the island from the peninsula, but the two have been connected during periods of lower sea level. The area of the island is approximately 2000 km^2. Fieldwork was carried out near the M.V. Popov Polar Meteorological Station in the northwest corner of the island (73°19’ N, 70°03’ E). The station has been occupied since 1933 and has been used as a base for a variety of purposes including meteorological and oceanographic observations, hydrocarbon exploration, military operations, and atmospheric studies using rockets. Permafrost thickness of the marine sediments of the modern coastal wetlands is 30 m on the average, varying from 2–10 meters at the coastline and 50–80 m inland. Permafrost thickness averages approximately 125 m on Terrace I, and approximately 240 m on Terrace II (Orekhov, Slagoda, & Popov, 2017; Trofimov, 1975).

We recorded 65 species vascular plant species in the vicinity of the Popov station. The only other records from the island were by F.R. Kielling, who recorded 17 species during the Vega Expedition of A.E. Nordensköld (1878–79), and Olga Rebristatya, who recorded 75 species of vascular plants in the southeast part of the island (Rebristaya 1995).

The main part of the ground observations at Ostrov Belyy were conducted at two study sites: BO-1, loamy site (Fig. S3-2a and b), consists of zonal tundra. (Note the mistaken reversal of the B and O in our plot numbers for Ostrov Belyy). Zonal tundra on loamy soils is rare on the island because of the island's general wetness and abundance of sandy substrates. Reindeer, although not absent, are much less abundant than they are on the mainland. Small non-sorted circles (0.3–1 m diameters) (also called "frost boils") (Washburn, 1980) were common at BO-1, where we sampled plant communities of two microhabitats within the 5 x 5-m plots: BO-1a occurred in association with the moist graminoid-dominated habitats between non-sorted circles and BO-1b occurred on the more barren and drier centers of the circles.

BO-2, sandy site (Fig. S3-2c and d), is located on a low well-drained bluff of a small stream about two km southeast of the Popov station. Areas with sandy soils occur along most stream bluffs and lake margins that are relatively well-drained. The surfaces of many of these well-drained sites appear gray because of the low cover of vascular plants and high cover of the crustose liverwort *Gymnomitrion corallioides*. Similar habitats have recently been described in a study of mires on Ostrov Belyy (Makarova, Ermilov, Yurtaev, & Mansurov, 2015). Small non-sorted polygons, 10–30 cm in diameter, are abundant on this dry site, so we sampled the two main microhabitats of these polygons: BO-2a occurred in association with the dry *Gymnomitrion*-dominated habitats on the small-polygon centers, and BO-2b occurred in the mossy cracks between the polygons.

Kharasavey

a.

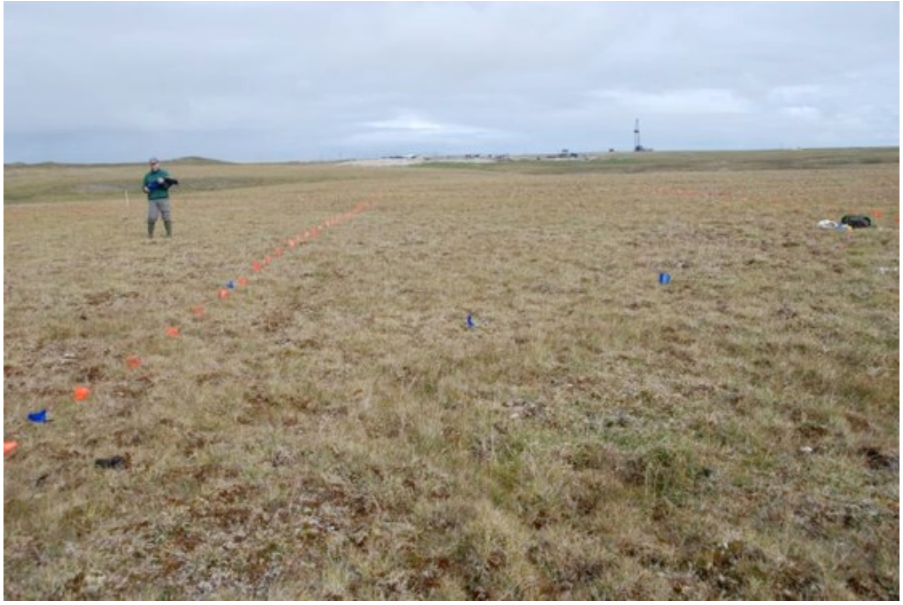

b.

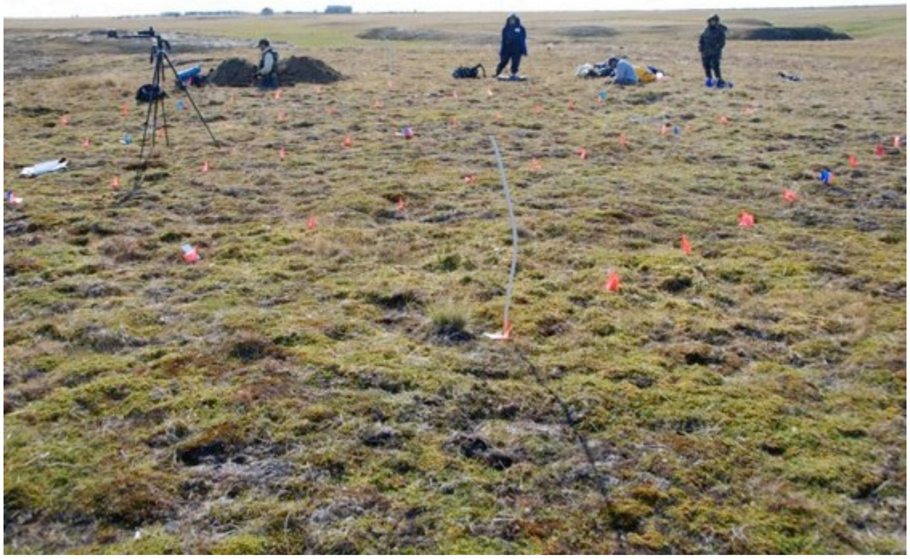

Figure S3-3. Vegetation of the Kharasavey (KH) study location. a. KH-1, loamy site. The vegetation is moist graminoid, prostrate dwarf-shrub, moss tundra, dominated by *Carex bigelowii, Calamagrostis holmii, Salix polaris, Dicranum elongatum* and *Cladonia* spp**. b.** KH-2b, sandy site. The vegetation is moist sedge, prostrate dwarf-shrub, moss tundra, dominated by *Carex bigelowii, Salix nummularia, Dicranum* sp., and *Cladonia* spp. (Photos: D.A. Walker.)

Kharasavey is located on the northwestern coast of the Yamal Peninsula (71°12' N, 66°56' E), approximately 60 km northwest of the Bovanenkovo gas field and 76 km northwest of Vaskiny Dachi. The area is part of a large complex of gas fields in west central Yamal. Pipelines and rail links are planned to Bovanenkovo. Kharasavey is also the endpoint of several reindeer-herd migration routes. Ongoing research at Bovanenkovo, Laborovaya and other locations on the Yamal is examining the social-ecological consequences of gas development and adaptations by the local Nenetsy people (Forbes, 2013; Kumpula, Forbes, Stammler, & Meschtyb, 2012). The flat to undulating local terrain is derived from sediments of marine terraces I and II (see Supplemental Information, Appendix S1). The terrace surfaces are relatively well drained and highly dissected by many small gullies and drainages. The drainages are continually expanding and growing due to erosion by cryogenic landslides of the underlying massive ground ice. Thermokarst features, including thaw lakes, drained thaw lakes, small thermokarst ponds, and ice-wedge polygons, are common in nearby peaty lowlands of the larger streams and rivers in the vicinity of Kharasavey.

There is no long-term climate record from Kharasavey. The nearest comparable coastal weather station is approximately 100 km south at Mare-Sale, where the mean annual air temperature (1961-1990) prior to the recent warming trend was -8.5 ˚C. The mean July temperature for the same period was 7.2 ˚C, and the air temperature summer warmth index (SWI_a) was 19.6 ˚C mo. The satellite-derived summer warmth index of the ground surface (SWI_g, 1982-2003) is 18.52 ˚C mo. The mean annual precipitation is 298 mm. The known local Kharasavey flora consists of 125 species of vascular plants (Rebristaya et al., 1995).

Four study sites were established at Kharasavey. KH-1, loamy site (Fig. S3-3a), is located on a homogeneous portion of terrace II with silt-loam soils. Common plants on the upland tundra areas include dwarf shrubs (e.g., *Salix polaris, S. lanata,* and *S. glauca*), graminoids (e.g, *Carex bigelowii, Calamagrostis holmii, Arctagrostis latifolia, Eriophorum angustifolium, Alopecurus alpinus, Poa arctica,* and *Luzula confusa*), forbs (e.g., *Saxifraga cernua, S. foliolosa,* and *Rumex arcticus)*, mosses (e.g., *Dicranum elongatum, Polytrichum strictum, Aulacomnium* spp*.,* and *Hylocomium splendens*), and lichens (e.g., *Cladonia* spp*., Sphaerophorus globosus, Peltigera aphthosa, Thamnolia subuliformis,* and *Cetraria* spp.). Large areas with sandy soils were uncommon. Small dune-like remnant sandy features occur along some of the creeks, but no extensive sandy areas with sufficient flat homogeneous terrain for a 50 x 50-m grid could be located. Consequently, we selected two 10 x 10-m sandy areas along bluffs of two small streams and supplemented the vegetation data with another nearby 5 x 5-m plot. Kharasavey-2a (KH-2a, sandy site) is on a small bluff of terrace I adjacent to a creek with thin sands over much of the grid. KH-2b, sandy site (Fig. S3-3b), is on a sandy portion of terrace II, where the vegetation is dominated by *Carex bigelowii, Salix nummularia, Dicranum* spp., and *Cladonia* spp. Sites KH-2a and KH-2b had two plots each. The fifth sandy plot (KH-RV-49) was located on an adjacent sandy feature near site KH-2b.

Vaskiny Dachi

a.

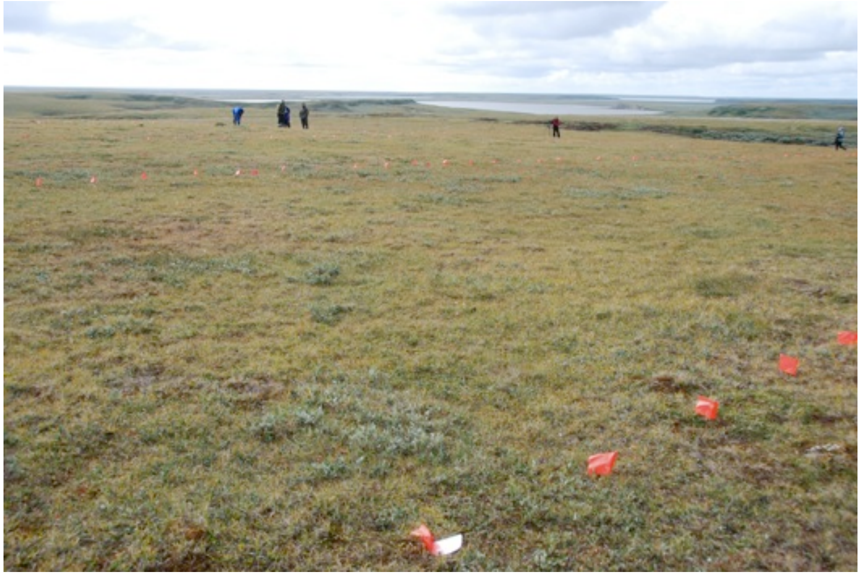

b.

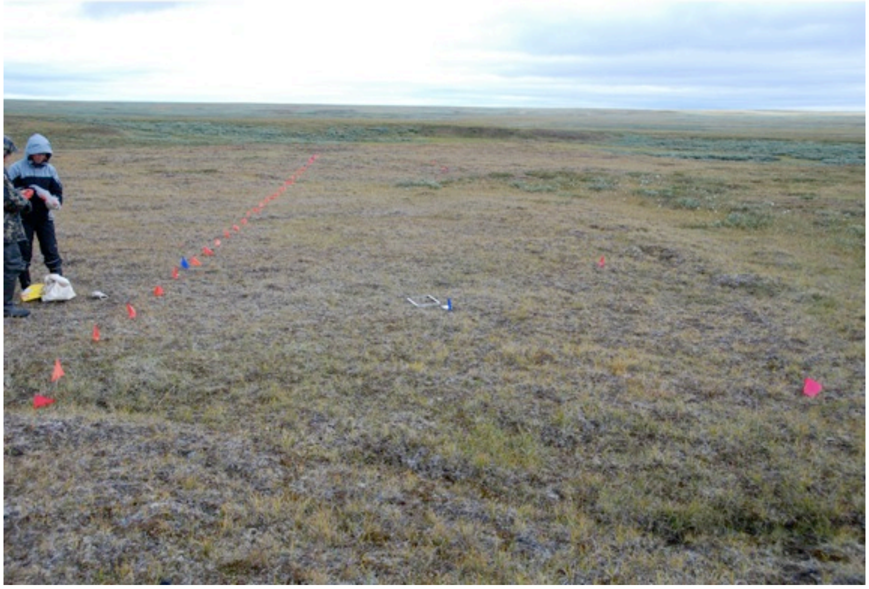

Figure S3-4. Vegetation of the Vaskiny Dachi EAT study location. a. VD-1, loamy site, on Terrace IV. The vegetation is heavily grazed sedge, dwarf-shrub, moss tundra dominated by *Carex bigelowii, Vaccinium vitis-idaea, Salix glauca, Hylocomium splendens,* and *Aulacomnium turgidum*. **b.** VD-3, sandy site, on Terrace II. The vegetation is a dry dwarf-shrub, lichen tundra dominated by *Carex bigelowii, Vaccinium vitis-idaea, Cladonia arbuscula, Sphaerophorus globosus, Racomitrium lanuginosum,* and *Polytrichum strictum*. (Photos: D.A. Walker.)

The location is approximately 1.4 km west of the Obskaya-Bovanenkovo railroad and 21 km east-southeast of the main airfield at the Bovanenkovo gas field, the largest gas field on the peninsula. The Vaskiny Dachi Research Station (VD) was established in 1988 to support Russian Academy of Science research associated with railroad construction and gas-field development on the Yamal

Peninsula. Since 1993 it has been a major focus of research for the Circumpolar Active Layer Monitoring (CALM) project of Dr. Marina Leibman and colleagues from the Earth Cryosphere Institute (Leibman, Gubarkov, & Khomutov, 2012; Leibman, Khomutov, Gubarkov, Mullanurov, & Dvornikov, 2015).

Gentle hilly terrain is associated with a series of highly eroded marine terraces of various ages and floodplains of the widely meandering Se-Yakha and Mordy-Ykha rivers. Despite the long period of research, there is no climate station at Vaskiny Dachi because of difficulties associated with maintaining a site with the annual reindeer migrations through the area. The mean annual summer warmth index (SWI_g) derived from satellite data is 29.6 ˚C mo.

The Vaskiny Dachi area is within Arctic bioclimate subzone D. The landslides and associated botanical features of the central Yamal have received considerable attention (Ermokhina, 2009; Rebristaya & Khitun, 1998; Rebristaya, VV, Chernyadjeva, & Leibman, 1995). A striking aspect of the regional vegetation is the abundance of willow thickets (*Salix lanata, S. glauca*), which cover many hill slopes and valley bottoms in association with the landslides (Rebristaya & Khitun, 1998; Ukraintseva, 2008; Ukraintseva, Leibman, & Streletskaya, 2000; Ukraintseva, Streletskaya, Ermokhina, & Yermakov, 2003).

Three study sites were established at Vaskiny Dachi in stable areas that avoided the landslides. VD-1 (Fig. S3-4a) and VD-2, loamy sites, are on gentle hills associated with marine terraces III and IV (Kazantsevskaya and Ermanovsky-age). The soils are silt loams. The vegetation of VD-1 and VD-2 is heavily grazed sedge, dwarf-shrub-moss tundra, dominated by *Carex bigelowii, Vaccinium vitis-idaea, Salix glauca, Hylocomium splendens,* and *Aulacomnium turgidum* at VD-1, and by *Betula nana, Calamagrostis holmii,* and *Aulacomnium turgidum* at VD-2.

VD-3, sandy site (Fig. S3-4b), is on a more recent fluvial terrace (terrace II, Table 1), comprised of finely interbedded sandy, silty, loamy, and organic layers of several millimeters to several centimeters thick. Vegetation at this site is prostrate dwarf-shrub, sedge, lichen tundra dominated by *Vaccinium vitis-idaea, Cladonia arbuscula,* and *Racomitrium lanuginosum*.

Laborovaya

a.

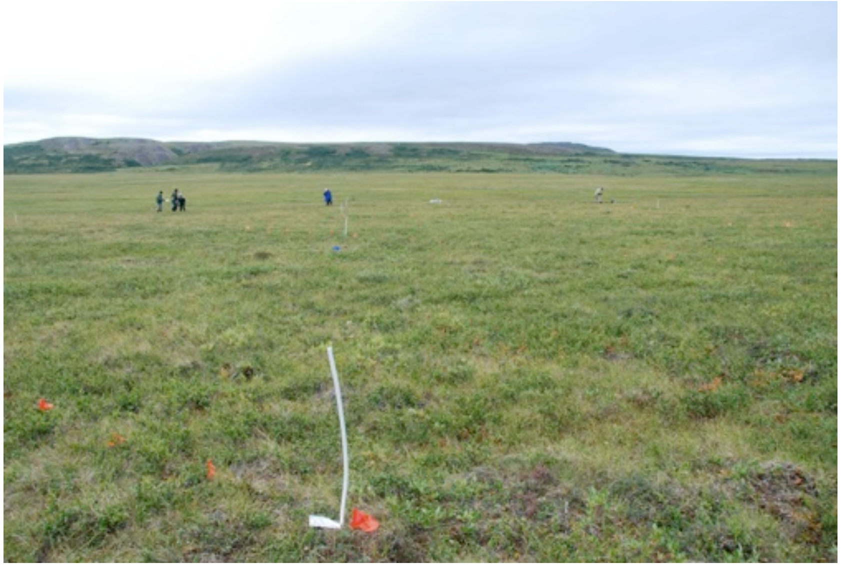

b.

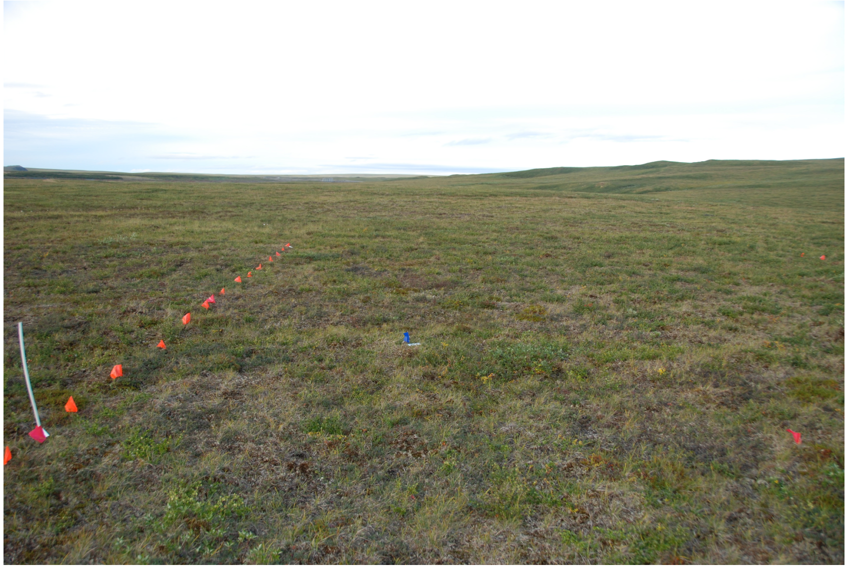

Figure S3-5. Vegetation of the Laborovaya EAT study location. a. LA-1, loamy site. The vegetation is a moist dwarf-shrub, sedge, moss tundra dominated by *Betula nana, Vaccinium vitis-idaea, V. uliginosum, Carex bigelowii, Eriophorum vaginatum, Aulacomnium palustre, Hylocomium splendens, and Dicranum* spp. **b.** LA-2, sandy site. The vegetation is moist/dry dwarf-shrub, lichen tundra dominated by *Betula nana, Vaccinium vitis-idaea, V. uliginosum, Carex bigelowii, Cladonia arbuscula, Sphaerophorus globosus,* and *Polytrichum strictum*. (Photos: D.A. Walker.)

The Laborovaya location (67˚ 42' N, 68˚ 01' E) is in the foothills near the northern end of the Polar Urals, about 21 km northeast of the small settlement of Laborovaya at km 147 of the Obskaya-Paijuta railway/ road corridor. The local physiography consists of flat plains with thaw lakes to the east and north. Hills with glaciated sandstone bedrock outcrops occur to the west and south. Surface sediments on the plains consist primarily of Pleistocene sands underlain by saline silts and

clays, similar to the geological situation on the main part of the Yamal Peninsula. Laborovaya and all EAT locations north of here lie within the continuous permafrost zone. This is a long-term study location of Dr. Bruce Forbes and researchers at the Arctic Center, Rovaniemi, Finland (Forbes, 1997).

The nearest year-round meteorological station is at Salekhard, 150 km to the south, near the mouth of the Ob River, which is not comparable because Salekhard is in the forest and is warmer and calmer than the Laborovaya region, which is strongly affected by its proximity to the Polar Urals. The mean satellite-derived summer-warmth index (SWI_g) for Laborovaya is 36.6 ˚C mo.

Phytogeographically, the study site lies about 100 km north of the latitudinal treeline within the southern tundra subzone (Subzone E of the Circumpolar Arctic Vegetation Map). This area and all of the Yamal Peninsula is within the Yamal-Gydan West Siberian floristic subprovince, which is characterized by a low floristic richness due to gaps in the ranges of species with predominantly montane, east Siberian distributions and western (amphi-Atlantic) distributions (Yurtsev, 1994). The region's vegetation has been mapped and described at small scale according to the Russian approach to vegetation classification (Ilyina, Lapshina, Makhno, Meltzer, & Romanova, 1976; Meltzer, 1984). Ridge tops on the sandstone hills are dry. Well-developed stands of green alder (*Alnus viridis*) are common on south-facing slopes and especially in riparian floodplains. Shrub willows (*Salix* spp.) are generally <30 cm tall in open tundra situations, but individuals >2 m tall occur on riparian floodplains and south-facing hill slopes. The areas between hills are a mix of wetlands and mesic tundra vegetation. The study area is extensively grazed in summer by reindeer herds belonging to the Yamal Nenetsy people.

Two study sites were established at Laborovaya. LA-1, loamy site (Fig. S3-5a), is located in a valley between two sandstone ridges. The tundra on moist silt-loam soils is dominated by dwarf-shrubs (*Betula nana, Vaccinium vitis-idaea,* and *V. uligino*sum), sedges (*Carex bigelowii,* and *Eriophorum vaginatum*), and mosses (*Aulacomnium palustre, Hylocomium splendens,* and *Dicranum* spp.)

LA-2, sandy site (Fig. S3-5b), is on a younger (somewhat drier sandy terrace of a small stream with tundra consisting mainly of dwarf shrubs (*Betula nana, Vaccinium vitis-idaea,* and *V. uliginosum*) and lichens (*Cladonia arbuscula* and *Sphaerophorus globosus*).

Nadym

a.

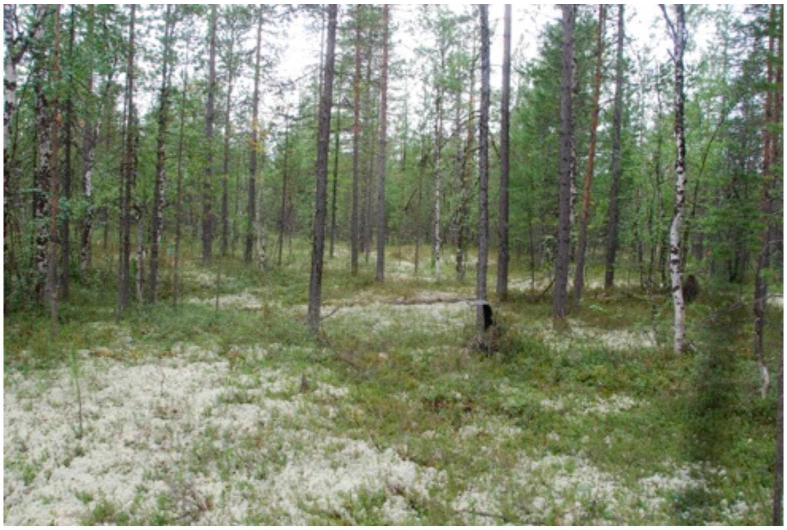

b.

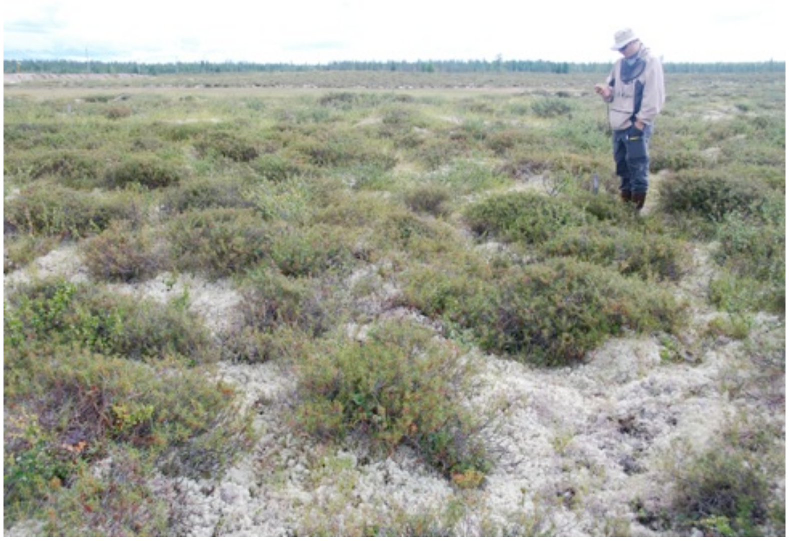

Figure S3-6. Vegetation of the Nadym EAT study location. a. ND-1, sandy forest site. The trees are mainly Scots pine (*Pinus sylvestris*), and mountain birch (*Betula tortuosa*) mixed with Siberian larch (*Larix sibirica*). The understory consists of dwarf shrubs (*Rhododendron tomentosum, Betula nana, Empetrum nigrum, Vaccinium uliginosum,* and *V. vitis-idaea*), lichens (mainly *Cladonia stellaris*) and mosses (mainly *Pleurozium schreberi*). (Photo: P. Kuss). **b.** ND-2, loamy tundra site. Hummocky tundra consists of a complex of vegetation with a *Rhododendron tomentosum-Betula nana-Cladonia* spp. dwarf-shrub community on the hummocks and a *Cladonia stellaris-Carex glomerata* lichen community in the inter-hummock areas. (Photo: D.A. Walker.)

The southernmost EAT location (65˚ 19’ N, 72˚ 53’ E) is about 30 km south-southeast of the city of Nadym, in the forest-tundra transition of the Boreal bioclimate zone. The climate is influenced by maritime air masses from the Atlantic Ocean and continental air masses from central Asia. The mean annual temperature is -5.9 ˚C; the mean July temperature is 15.8 ˚C, and the mean SWI_g is 41.3 ˚C mo. Mean annual precipitation is 478 mm with over half of the total (252 mm) occurring during the summer (June-August). This is the only EAT location in the discontinuous permafrost zone; all others are in the continuous permafrost zone (Brown, Ferrians, Heginbottom, & Melnikov, 1997). It is also the only EAT location with forests and the only location along the mainland portion of the EAT that is not heavily grazed by reindeer, mainly because the local network of oilfield infrastructure limits access by herders and their animals. This is a long-term study area of Natalia Moskalenko and other researchers from the Earth Crysophere Institute (Melnikov, Leibman, Moskalenko, & Vasiliev, 2004; Moskalenko, 2003; 2007).

Two study sites were examined: ND-1, loamy, forest site (Fig. S3-6a) is on a lower and relatively young (about 20–40 ka BP) terrace of the Nadym River with no peat or permafrost. The vegetation at ND-1 is open birch-pine (*Pinus sylvestris* and *Betula tortuosa*) woodland with a dwarf-shrub and lichen understory (*Betula nana, Rhododendron tomentosum,* and *Cladonia stellaris*).

ND-2, sandy tundra site (Fig. S3-6b), is on a higher and relatively old (60–80 ka BP) fluvial-lacustrine plain. The vegetation at ND-2 consists of hummocky tundra with a complex of two dominant plant communities. A dwarf-shrub and lichen community (*Rhododendron tomentosum, Betula nana,* and *Cladonia* spp.) occurs on the tops of small earth hummocks and a lichen community (*Cladonia stellaris,* and *Carex glomerata*) occurs in the inter-hummock areas (Moskalenko, 2008). The ND-2 study plots were located around the margins of the Nadym, West Siberia 100 x 100-m Circumpolar Active Layer Monitoring (CALM) grid to avoid disturbance to the central monitoring areas of the grid.

Literature cited

Alexandrova, V. D. (1980). The Arctic and Antarctic: Their Division into Geobotanical Areas. Cambridge: Cambridge University Press.

Brown, J., Ferrians, O. J., Heginbottom, J. A., & Melnikov, E. S. (1997). *Circum-Arctic Map of Permafrost and Ground-Ice Conditions*. *U.S. Geological Survey*. U.S. Geological Survey Map CP-45.

CAVM Team, Gould, W. A., Bliss, L. C., Edlund, S. A., Raynolds, M. K., Zoltai, S. C., et al. (2003). Circumpolar Arctic Vegetation Map. *Conservation of Arctic Flora and Fauna Map (CAFF) Map No. 1*. Anchorage, AK: U.S. Fish and Wildlife Service.

Dibner, V. D. (1965). The history of late Pleistocene and Holocene sedimentation in Franz Josef Land (in Russian). *Transactions of the Scientific Research Institute of the Geology of the Arctic*, *143*, 300–318.

Ermokhina, K. A. (2009). *Phytoindication of exogenic processes in Central Yamal tundra*. Moscow University, Moscow.

Forbes, B. C. (1997). Tundra disturbance studies IV. Species establishment on anthropogenic primary surfaces, Yamal Peninsula, Northwest Siberia, Russia. *Polar Geography*, *21*(1), 79–100.

Forbes, B. C. (2013). Cultural resilience of social-ecological systems in the Nenets and Yamal-Nenets Autonomous Okrugs, Russia: a focus on reindeer nomads of the tundra. *Ecology and Society*, *18*(4), art36. http://doi.org/10.5751/ES-05791-180436

Forman, S. L., Lubinski, D. J., Zeeberg, J. J., Snyder, J. A., Siegert, M. J., & Matishov, G. G. (2004). A review of postglacial emergence on Svalbard, Franz Josef Land and Novaya Zemlya, northern Eurasia. *Quaternary Science Reviews*, *23*(11-13), 1391–1434. http://doi.org/10.1016/j.quascirev.2003.12.007

Ilyina, I. S., Lapshina, E. I., Makhno, V. D., Meltzer, L. I., & Romanova, E. A. (1976). Vegetation of the West Siberian Plain, (1:1,500,000-scale map, 4 sheets). Moscow: State Cartography Press (GUGK).

Koryakin, Y. V., & Shipilov, E. V. (2009). Geochemical specifics and 40Ar/39Ar age of the basaltoid magmatism of the Alexander Land, Northbrook, Hooker and Hayes Islands (Franz Josef Land Archipelago) (In Russian). *Doklady Earth Sciences*, *425*, 260–263..

Kumpula, T., Forbes, B. C., Stammler, F., & Meschtyb, N. (2012). Dynamics of a coupled system: multi-resolution remote sensing in assessing social-ecological responses during 25 Years of gas field development in arctic Russia. *Remote Sensing*, *4*(4), 1046–1068. http://doi.org/10.3390/rs4041046

Leibman, M. O., Gubarkov, A. A., & Khomutov, A. V. (2012). Research Station Vaskiny Dachi: TICOP Excursion Guidebook: Tenth International Conference on Permafrost TICOP: Resources and risks of permafrost areas in a changing world (pp. 1–50). Tyumen: Pechatnik, Russia: North Press.

Leibman, M., Khomutov, A., Gubarkov, A., Mullanurov, D., & Dvornikov, Y. (2015). The research station “Vaskiny Dachi,” Central Yamal, West Siberia, Russia - A review of 25 years of permafrost studies. *Fennia*, *193*, 3–30.

Lubinski, D. L., Forman, S. L., & Miller, G. H. (1999). Holocene glacier and climate fluctuations on Franz Josef Land, Arctic Russia, 80˚N. *Quaternary Science Reviews*, *18*, 85–108.

Makarova, O. L., Ermilov, S. G., Yurtaev, A. A., & Mansurov, R. I. (2015). The first data on the soil mites (Acari) of the Arctic Belyi Island (Northern Yamal, the Kara Sea). *Entomological Review*, *95*(6), 805–810. http://doi.org/10.1134/S0013873815060147

Melnikov, E. S., Leibman, M. O., Moskalenko, N. G., & Vasiliev, A. A. (2004). Active layer monitoring in West Siberia. *Polar Geography*, *28*(4), 284.

Meltzer, L. I. (1984). Zonal division of tundra vegetation of the West Siberian plain. (In Russian). In *Vegetation of western Siberia and its mapping* (pp. 7–19). Novosibirsk: Akademia Nauk.

Moskalenko, N. G. (2003). Interactions between vegetation and permafrost on some CALM grids in Russia (Vol. 2, pp. 789–794). Presented at the Permafrost: Proceedings of the Eighth International Conference on Permafrost, Zurich, Switzerland: A.A. Balkema Publishers.

Moskalenko, N. G. (2007). Impact of climate changes on West Siberia northern taiga ecosystems. (pp. 98–99). Presented at the Proceedings of the VIII International Symposium on Cold Region Development, Tampere, Finland.

Moskalenko, N. G. (2008). Overview of vegetation dynamics, disturbance and recovery studies in Nadym and Yamal areas. Presented at the Yamal Land-Cover Land-Use Change Workshop, Moscow, Russia.

Orekhov, P., Slagoda, E., & Popov, K. (2017). Factors of spatial differentiation of cryogenic geosystems of the Belyy Island Arctic tundras. Presented at the Arctic Science Summit Week, Prague, 31 Mar-07 Apr, Prague.

Rebristaya, O. V., & Khitun, O. V. (1998). Botanical-geographical features of the central Yamal flora. (In Russian). *Botanicheskii Zhurnal*, *83*(7), 37–52.

Rebristaya, O. V., (1995). Vascular plants of Belyy Island (Kara Sea). (In Russian). *Botanchisky Zhurnal*, *80*, 26–36.

Rebristaya, O. V., VV, K. O., Chernyadjeva, I. V., & Leibman, M. O. (1995). Dynamics of vegetation on the cryogenic landslips in the central part of the Yamal Peninsula. *Botanchisky Zhurnal*, *80*(4), 31–47.

Trofimov, V. T. (1975). Polustrov Yamal (Yamal Peninsula). (In Russian).Moscow: Moscow University Press.

Ukraintseva, N. G. (2008). Vegetation response to landslide spreading and climate change in the West SiberianTundra. In *Ninth International Conference on Permafrost* (pp. 1793–1798). Fairbanks.

Ukraintseva, N. G., Leibman, M. O., & Streletskaya, I. D. (2000). Peculiarities of landslide process in saline frozen deposits of central Yamal, Russia. In *Landslides. Proceedings VIII International Symposium on Landslides 3* (pp. 1495–1500). London: Thomas Telford.

Ukraintseva, N. G., Streletskaya, I. D., Ermokhina, K. A., & Yermakov, S. Y. (2003). Geochemical properties of plant-soil-permafrost system at landslide slopes, Yamal, Russia. In *Proceedings of the International Conference on Permafrost, Zurich, 21-25 July 2003* (Vol. II, pp. 1149–1154). Lisse, Nethrerlands: A.A. Balkema, Publishers.

Walker, D. A., Carlson, S., Frost, G. V., Matyshak, G. V., Leibman, M. E., Orechov, P., et al. (2011). *2010 Expedition to Krenkel Station, Hayes Island, Franz Josef Land Russia*. *AGC Data Report*. Fairbanks, AK: University of Alaska Fairbanks.

Walker, D. A., Epstein, H. E., Leibman, M. E., Moskalenko, N. G., Kuss, H. P., Matyshak, G. V., et al. (2008). *Data report of the 2007 Yamal expedition to Nadym, Laborovaya, and Vaskiny Dachi, Yamal Peninsula region, Russia*. *AGC Data Report*.

Walker, D. A., Epstein, H. E., Leibman, M. E., Moskalenko, N. G., Kuss, J. P., Matyshak, G. V., et al. (2009a). *Data Report of the 2007 and 2008 Yamal Expeditions: Nadym, Laborovaya, Vaskiny Dachi, and Kharasavey*. *AGC Data Report* (p. 133). Fairbanks, AK: University of Alaska.

Walker, D. A., Orekhov, P., Frost, G. V., Matyshak, G., Epstein, H. E., Leibman, M. O., et al. (2009b). *The 2009 Yamal Expedition to Ostrov Belyy and Kharp, Yamal Region, Russia*. *AGC Data Report* (p. 63). Fairbanks, AK: University of Alaska Fairbanks.

Washburn, A. L. (1980). Geocryology: A Survey of Periglacial Processes and Environments. New York: Halsted Press, John Wiley and Sons.

Yurtsev, B. A. (1994). The floristic division of the Arctic. *Journal of Vegetation Science*, *5*(6), 765–776.
