# Supplementary material for: Vegetation on mesic loamy and sandy soils along a 1700‐km maritime Eurasia Arctic Transect
Source: Appl Veg Sci. 2019 Feb 27;22(1):150–67. doi: 10.1111/avsc.12401 (PMC6519894; doi:10.1111/avsc.12401)
Supplement: Supplementary file 1 — Appendix S1. Geological setting of the Yamal Peninsula. Appendix S2. Typical plot layout. Appendix S3. Eurasia Arctic Transect location and site descriptions. Appendix S4. Eurasia Arctic Transect species cover‐abundance data. Appendix S5. Eurasia Arctic Transect environmental data. Appendix S6. Full synoptic table. Appendix S7. Diagnostic, constant, and dominant taxa for EAT clusters. Appendix S8. Trends of selected soil and vegetation properties vs. summer warmth index. Appendix S9. Regression equations for trend lines of analysed variables. Appendix S10. Number of species per plot along the Eurasia Arctic Transect. Appendix S11. Correlations between four axes of the DCA ordination and environmental variables. Appendix S12. Lichen‐rich tundra of Hayes Island. [file AVSC-22-150-s001.zip › supinfo/Appendix_S5_Table_5.2_Codes_for_environmental_variables_20190210.pdf]

Supporting Information Appendix S5, Table S5.1 Field codes for environmental variables, Eurasia Arctic Transect.

<u>Landforms (Code)</u>
1 Hills (including kames and moraines)
2 Talus slope
3 Colluvial basin
4 Glaciofluvial and other fluvial terraces
5 Marine terrace
6 Floodplains
7 Drained lakes and flat lake margins
8 Abandoned point bars and sloughs
9 Estuary
10 Lake or pond
11 Stream
12 Sea bluff
13 Lake bluff
14 Stream bluff
15 Sand dunes
16 Beach
17 Disturbed
18 Alluvial plain/abandoned
19 Island
20 Plain - residual surface
21 Marine terrace

<u>Surficial Geology/ Parent Material (Code)</u>
1 Glacial tills
2 Glaciofluvial deposits
3 Active alluvial sands
4 Active alluvial gravels
5 Stabilized alluvium (sands & gravels)
6 Undifferentiated hill slope colluvium
7 Basin colluvium and organic deposits
8 Drained lake or lacustrine organic deposits
9 Lake or pond organic, sand, or silt
10 Undifferentiated sands
11 Undifferentiated clay
12 Roads and gravel pads
13 Loess
14 Fine sand
15 Marine sands
16 Marine clay

<u>Surficial Geomorphology/ Periglacial features (Code)</u>
1 Frost scars
2 Wetland hummocks
3 Turf hummocks
4 Gelifluction features
5 Strangmoor or aligned hummocks
6 High- or flat-centered polygons
7 Mixed high- and low-centered polygons
8 Sorted and non-sorted stripes
9 Palsas
10 Thermokarst pits
11 Featureless or with less 20% frost scars
12 Well-developed hillslope water tracks and small streams > 50 cm deep
13 Poorly developed hillslope water tracks, < 50 cm deep
14 Gently rolling or irregular microrelief
15 Stoney surface
16 Lakes and ponds
17 Disturbed
18 Hillslope hummocks
19 Wetland
20 Small non-sorted polygon

<u>Microsites (Code)</u>
1 Frost-scar element
2 Inter-frost scar element
3 Strang or hummock
4 Flark, interstrang, or interhummock area
5 Polygon center
6 Polygon trough
7 Polygon rim
8 Stripe element
9 Inter-stripe element
10 Point bar (raised element)
11 Slough (wet element)
12 Raised ring of non-sorted circle
13 Thermokarst pit
14 Tops of small non-sorted polygons
15 Cracks between small non-sorted polygons

<u>Site Moisture (modified from Komárková 1983) (Scalar)</u>
1 Extremely xeric - almost no moisture; no plant growth
2 Very xeric - very little moisture; dry sand dunes
3 Xeric - little moisture; stabilized sand dunes, dry ridge tops
4 Subxeric - noticeable moisture; well-drained slopes, ridges
5 Subxeric to mesic - very noticeable moisture; flat to gently sloping
6 Mesic-moderate moisture; flat or shallow depressions
7 Mesic to subhygric - considerable moisture; depressions
8 Subhygric - very considerable moisture; saturated but with < 5% standing water < 10 cm deep
9 Hygric - much moisture; up to 100% of surface under water 10 to 50 cm deep; lake margins, shallow ponds, streams
10 Hydric - very much moisture; 100% of surface under water 50 to 150 cm deep; lakes, streams

<u>Soil Moisture (from Komárková 1983) (Scalar)</u>
1 Very dry - very little moisture; soil does not stick together
2 Dry - little moisture; soil somewhat sticks together
3 Damp - noticeable moisture; soil sticks together but crumbles
4 Damp to moist - very noticeable moisture; soil clumps
5 Moist - moderate moisture; soil binds but can be broken apart
6 Moist to wet - considerable moisture; soil binds and sticks to fingers
7 Wet - very considerable moisture; water drops can be squeezed out of soil
8 Very wet - much moisture can be squeezed out of soil
9 Saturated - very much moisture; water drips out of soil
10 Very saturated - extreme moisture; soil is more liquid than solid

Topographic Position (Code)
1 Hill crest or shoulder
2 Side slope
3 Footslope or toeslope
4 Flat
5 Drainage channel
6 Depression
7 Lake or pond

Estimated Snow Duration (Scalar)
1 Snow free all year
2 Snow free most of winter; some snow cover persists after storm but is blown free soon after
3 Snow free prior to melt out but with snow most of winter
4 Snow free immediately after melt out
5 Snow bank persists 1-2 weeks after melt out
6 Snow bank persists 3-4 weeks after melt out
7 Snow bank persists 4-8 weeks after melt out
8 Snow bank persists 8-12 weeks after melt out
9 Very short snow free period
10 Deep snow all year

Animal and Human Disturbance (degree) (Scalar)
0 No sign present
1 Some sign present; no disturbance
2 Minor disturbance or extensive sign
3 Moderate disturbance; small dens or light grazing
4 Major disturbance; multiple dens or noticeable trampling
5 Very major disturbance; very extensive tunneling or large pit

Animal and Human Disturbance (type) (Code)
1 Ptarmigan scat
2 Caribou tracks
3 Caribou scat
4 Goose tracks, scat, grazing
5 Squirrel mounds
6 Vole tracks & scat
7 Vehicle tracks
8 Fox seat

Stability (Scalar)
1 Stable
2 Subject to occasional disturbance
3 Subject to prolonged but slow disturbance such as solifluction
4 Annually disturbed
5 Disturbed more than once annually

Exposure Scale (Scalar)
1 Protected from winds
2 Moderate exposure to winds
3 Exposed to winds
4 Very exposed to winds

Texture (USDA Soil Survey) (Code)
1 Sand
2 Loamy sand
3 Sandy loam
4 Sandy clay loam
5 Sandy clay
6 Loam
7 Clay loam
8 Clay
9 Silty clay
10 Silty clay loam
11 Silt loam
12 Silt
13 Peat
