# Supplementary material for: Vegetation on mesic loamy and sandy soils along a 1700‐km maritime Eurasia Arctic Transect
Source: Appl Veg Sci. 2019 Feb 27;22(1):150–67. doi: 10.1111/avsc.12401 (PMC6519894; doi:10.1111/avsc.12401)
Supplement: Supplementary file 1 — Appendix S1. Geological setting of the Yamal Peninsula. Appendix S2. Typical plot layout. Appendix S3. Eurasia Arctic Transect location and site descriptions. Appendix S4. Eurasia Arctic Transect species cover‐abundance data. Appendix S5. Eurasia Arctic Transect environmental data. Appendix S6. Full synoptic table. Appendix S7. Diagnostic, constant, and dominant taxa for EAT clusters. Appendix S8. Trends of selected soil and vegetation properties vs. summer warmth index. Appendix S9. Regression equations for trend lines of analysed variables. Appendix S10. Number of species per plot along the Eurasia Arctic Transect. Appendix S11. Correlations between four axes of the DCA ordination and environmental variables. Appendix S12. Lichen‐rich tundra of Hayes Island. [file AVSC-22-150-s001.zip › supinfo/Appendix_S6_Synoptic_Table_20190210.pdf]

Supporting Information, Appendix S6. Full synoptic table for statistical clusters of mesic tundra vegetation plots along the Eurasia Arctic Transect. Values are frequency of the given plant taxon within the indicated cluster (see Fig. 3, main text). Fidelity of diagnostic species was calculated using the phi coefficient (Chytrý et al. 2002) for individual clusters compared to the full suite of clusters. Diagnostic taxa are ordered according to descending fidelity (modified phi values). Taxa with very high fidelity (modified phi ≥ 0.8) have frequency values highlighted in dark gray; those with high fidelity (modified phi > 50) are highlighted in light gray. The second column in the table contains the plant growth form for each species: **bl**, bryophyte, liverwort; **bma**, bryophyte, moss, acrocarpous; **bmp**, bryophyte, moss, pleurocarpous; **bms**, bryophyte, moss, sphagnoid; **fe**, forb, erect; **fm**, forb, mat, cushion or rosette; **gs**, graminoid, sedge; **gg**, graminoid, grass; **gr**, graminoid, rush; **lc**, lichen, crustose; **lfo**, lichen, foliose; **lfr**, lichen, fruticose; **sle**, shrub, low, evergreen; **sld**, shrub, low, deciduous; **sde**, shrub, dwarf, evergreen; **sdd**, shrub, dwarf, deciduous; **tne**, tree, needleleaf, evergreen; **tnd**, tree, needleleaf, deciduous; **tbd**, tree, broadleaf, deciduous; **vs**, vascular plant, seedless.

**Cluster nr.**		**1**	**2**	**4**	**5**	**6**	**7**	**3**
**Subzone(s) (soil texture)**		**FT(lom)**	**FT(snd)**	**E+D(snd)**	**D(lom)+C**	**B(lom)**	**B(snd)**	**A**
**Nr of relevés**		**5**	**6**	**15**	**20**	**10**	**10**	**10**
**Diagnostic taxa for cluster 1:**	**Growth form**							
Pinus sylvestris	tne	100	.	.	.	.	.	.
Betula pubescens	tbd	100	.	.	.	.	.	.
Larix sibirica	tnd	100	.	.	.	.	.	.
Vaccinium myrtillus	sdd	100	.	.	.	.	.	.
Juniperus communis	sle	80	.	.	.	.	.	.
Peltigera malacea	lfo	60	.	.	.	.	.	.
Pleurozium schreberi	bmp	100	17	47	5	.	.	.
Peltigera leucophlebia	lfo	100	.	13	50	20	.	.
Cladonia stellaris	lfr	100	83	20	.	.	.	.
Empetrum nigrum	sde	100	17	80	10	.	.	.
Vaccinium uliginosum	sdd	100	33	67	15	.	.	.
**Diagnostic taxa for cluster 2;**								
Carex globularis	gs	.	100	.	.	.	.	.
Andromeda polifolia	sde	.	83	7	.	.	.	.
Rubus chamaemorus	sdd	.	83	7	.	.	.	.
Rhododendron tomentosum s. *tomentosum*	sle	100	100	73	.	.	.	.
**Diagnostic taxa for cluster 4:**								
Flavocetraria nivalis	lfr	.	.	93	25	.	.	.
Salix phylicifolia	sld	.	.	67	10	.	.	.
Eriophorum vaginatum	gs	.	17	87	25	.	.	.
Pedicularis labradorica	fe	.	.	53	.	.	.	.
Asahinea chrysantha	lfr	.	.	40	.	.	.	.
Pertusaria dactylina	lc	.	.	47	.	.	10	.
Cladonia grayi	lfr	.	.	40	5	.	.	.
Schljakovia kunzeana	bl	.	.	33	.	.	.	.

Luzula wahlenbergii	gr	.	.	33	.	.	.	.
**Diagnostic taxon for clusters 5 & 6:**								
Arctagrostis latifolia	gg	.	.	20	95	100	10	.
**Diagnostic taxa for cluster 5:**								
Lophozia ventricosa	bl	.	.	40	80	.	.	.
Alopecurus borealis	gg	.	.	.	60	.	.	10
Salix reptans	sdd	.	.	13	55	.	.	.
Eriophorum angustifolium	gs	.	.	27	60	.	.	.
Tephroseris atropurpurea	fe	.	.	7	45	.	.	.
Peltigera canina	lfo	.	.	.	35	.	.	.
Peltigera aphthosa	lfo	.	.	.	40	10	.	.
Lichenomphalia hudsoniana	lfo	.	.	.	30	.	.	.
**Diagnostic taxa for cluster 6:**								
Blepharostoma trichophyllum	bl	.	.	.	5	100	.	.
Salix polaris	sdd	.	.	.	50	100	.	.
Tomentypnum nitens	bmp	.	.	13	20	90	.	.
Dryas octopetala	sde	.	.	.	40	100	50	.
Poa arctica	gg	.	.	7	40	80	.	.
Juncus biglumis	gr	.	.	.	.	60	20	.
Bryum cyclophyllum	bma	.	.	.	.	40	.	.
Stellaria longipes	fe	.	.	.	25	60	.	.
Sphenolobus minutus	bl	.	.	73	80	100	20	.
**Diagnostic taxa for cluster 7:**								
Pogonatum dentatum	bma	.	.	13	.	.	80	.
Oxyria digyna	fm	.	.	.	.	.	80	20
Gymnomitrion corallioides	bl	.	.	33	25	10	100	.
Luzula confusa	gr	.	.	.	60	10	100	.
Salix nummularia	sdd	.	.	27	50	.	100	.
Lloydia serotina	fe	.	.	.	.	.	50	.
Solorina crocea	lfo	.	.	.	.	.	50	.
Polytrichum piliferum	bma	.	.	7	.	10	50	.
Pohlia crudoides	bma	.	.	7	.	.	40	.
Gowardia nigricans	lfr	.	.	40	60	20	90	.
**Diagnostic taxa for cluster 3:**								
Stellaria longipes taxon edwardsii	fe	.	.	.	.	.	.	100
Papaver dahlianum agg. (*P. cornwallisense*)	fm	.	.	.	.	.	.	100
Phippsia algida	gg	.	.	.	.	.	.	100
Cochlearia groenlandica	fm	.	.	.	.	.	.	100
Lecidea ramulosa	lc	.	.	.	.	.	.	100
Orthothecium chryseum	bmp	.	.	.	.	10	.	100

Cladonia pocillum	lfr	.	.	.	.	10	.	100
Cetrariella delisei	lfr	.	.	20	.	.	.	100
Cerastium nigrescens v. *laxum*	fm	.	.	.	.	.	.	80
Fulgensia bracteata	lc	.	.	.	.	.	.	80
Saxifraga cernua	fe	.	.	.	5	.	.	80
Draba subcapitata	fm	.	.	.	.	.	20	90
Cirriphyllum cirrosum	bmp	.	.	.	.	.	.	70
Cerastium regelii	fm	.	.	.	.	10	.	70
Encalypta alpina	bma	.	.	.	.	.	.	60
Solorina bispora	lfo	.	.	.	.	.	.	60
Bryum rutilans	bma	.	.	.	.	.	.	60
Saxifraga cespitosa	fm	.	.	.	.	.	.	60
Distichium capillaceum	bma	.	.	.	.	30	.	80
Cetraria aculeata	lfr	.	.	.	.	.	20	70
Pohlia cruda	bma	.	.	.	.	40	.	80
Gowardia arctica	lfr	.	.	.	.	.	.	50
Saxifraga oppositifolia	fm	.	.	.	.	.	.	50
Cladonia symphycarpia	lfr	.	.	.	.	.	.	50
Stereocaulon rivulorum	lfr	.	.	.	.	.	.	50
Polytrichastrum alpinum	bma	.	.	.	30	10	60	100
Bartramia ithyphylla	bma	.	.	.	.	.	10	50
Callialaria curvicaulis	bmp	.	.	.	.	.	.	40
Campylium stellatum v. *arcticum*	bmp	.	.	.	.	.	.	40
Ditrichum flexicaule	bma	.	.	.	5	40	.	70
Protopannaria pezizoides	lc	.	.	.	5	.	.	40

Nondiagnostic taxa occuring in more than one cluster:

Cladonia stygia	lfr	60	100	93	60	.	.	.
Betula nana	sld	100	50	80	45	.	.	.
Dicranum fuscescens	bma	40	17	20	15	.	.	.
Cladonia cornuta	lfr	20	17	33	20	.	.	.
Polytrichum commune	bma	60	.	20	5	.	.	.
Festuca ovina [s. ovina]	gg	20	.	27	30	.	.	.
Peltigera neckeri	lfo	20	.	27	5	.	.	.
Cetraria islandica	lfr	100	33	100	100	100	60	100
Cladonia arbuscula s. lat.	lfr	60	17	93	75	100	30	.
Cladonia rangiferina	lfr	60	17	60	50	90	30	.
Ptilidium ciliare	bl	20	17	100	60	90	10	.
Polytrichum strictum	bma	20	50	73	50	100	70	.
Dicranum acutifolium	bma	20	17	20	35	70	20	.

Peltigera scabrosa	lfo	20	.	33	60	40	10	.
Cladonia gracilis s. lat.	lfr	20	.	67	95	90	60	.
Vaccinium vitis-idaea	sde	100	100	87	60	.	20	.
Flavocetraria cucullata	lfr	.	50	100	100	40	20	100
Cetraria laevigata	lfr	.	67	20	10	20	.	.
Cladonia amaurocraea	lfr	.	100	80	90	100	20	.
Cladonia coccifera s. lat.	lfr	.	50	93	90	100	70	.
Pohlia nutans	bma	.	33	40	45	40	30	.
Dicranum elongatum	bma	.	33	87	95	100	50	.
Cladonia deformis	lfr	.	33	13	20	.	.	.
Aulacomnium turgidum	bmp	.	17	87	100	100	20	.
Cladonia bellidiflora	lfr	.	17	60	40	.	20	.
Ptilium crista-castrensis	bmp	.	17	7	.	.	.	.
Polytrichum jensenii	bma	.	17	13	.	.	.	.
Cladonia sulphurina	lfr	.	33	13	5	.	.	.
Cladonia macrophylla	lfr	.	17	7	5	.	.	.
Pedicularis lapponica	fe	.	.	33	5	.	.	.
Polytrichum hyperboreum	bma	.	.	33	15	.	.	.
Hierochloë alpina	gg	.	.	27	20	.	.	.
Cladonia squamosa s. lat.	lfr	.	.	27	15	.	.	.
Pertusaria geminipara	lc	.	.	20	5	.	.	.
Cladonia cenotea	lfr	.	.	13	5	.	.	.
Valeriana capitata	fe	.	.	7	20	.	.	.
Orthocaulis binsteadii	bl	.	.	7	20	.	.	.
Calliergon stramineum	bmp	.	.	7	5	.	.	.
Sphagnum girgensohnii	bms	.	.	7	5	.	.	.
Arctocetraria andrejevii	lfr	.	.	7	10	.	.	.
Peltigera frippii	lfo	.	.	7	10	.	.	.
Ceratodon purpureus	bma	.	.	7	10	.	.	.
Salix hastata	sld	.	.	7	20	.	.	.
Cladonia chlorophaea	lfr	.	.	7	30	.	.	.
Carex bigelowii	gs	.	.	100	100	100	.	.
Oncophorus wahlenbergii	bma	.	.	13	10	50	.	.
Psoroma hypnorum	lc	.	.	13	20	40	.	.
Tritomaria quinquedentata	bl	.	.	7	45	60	.	.
Dactylina arctica	lfr	.	.	93	100	80	40	.
Sphaerophorus globosus	lfr	.	.	93	95	100	100	.
Cladonia uncialis	lfr	.	.	87	75	100	60	.
Calamagrostis holmii	gg	.	.	87	95	100	40	.
Cladonia subfurcata	lfr	.	.	80	60	50	40	.

Racomitrium lanuginosum	bmp	.	.	73	70	50	90	.
Hylocomium splendens	bmp	.	.	67	100	100	50	.
Dicranum spadiceum	bma	.	.	53	70	20	20	.
Bryoria nitidula	lfr	.	.	33	5	10	40	.
Cladonia stricta s. lat.	lfr	.	.	20	10	10	10	.
Cetrariella fastigiata	lfr	.	.	13	10	20	10	.
Alectoria ochroleuca	lfr	.	.	53	35	.	60	.
Pertusaria panyrga	lc	.	.	13	5	.	20	.
Aulacomnium palustre	bmp	.	.	20	20	.	10	.
Bistorta vivipara	fe	.	.	7	40	.	40	.
Conostomum tetragonum	bma	.	.	27	.	20	20	.
Ochrolechia inaequatula	lc	.	.	13	65	30	.	30
Thamnolia vermicularis	lfr	.	.	100	100	100	100	100
Bryocaulon divergens	lfr	.	.	80	85	50	100	40
Sanionia uncinata	bmp	.	.	33	40	70	10	20
Stereocaulon alpinum	lfr	.	.	33	40	20	20	70
Ochrolechia frigida	lc	.	.	100	25	90	100	10
Cladonia pyxidata	lfr	.	.	20	20	40	.	10
Pogonatum urnigerum	bma	.	.	7	.	.	.	20
Baeomyces rufus	lfr	.	.	7	.	.	.	10
Lobaria linita	lfo	.	.	.	30	50	.	.
Tetraplodon mnioides	bma	.	.	.	5	10	.	.
Micranthes foliolosa	fm	.	.	.	5	10	.	.
Eriophorum scheuchzeri	gs	.	.	.	5	30	.	.
Nephroma expallidum	lfo	.	.	.	5	20	.	.
Luzula nivalis	gr	.	.	.	5	30	.	.
Pedicularis hirsuta	fe	.	.	.	40	.	60	.
Parmelia omphalodes s. lat.	lfo	.	.	.	35	50	60	10
Sagina nivalis	fm	.	.	.	.	20	10	.
Dactylina ramulosa	lfr	.	.	.	.	20	10	.
Anthelia juratzkana	bl	.	.	.	.	50	.	40
Bryoerythrophyllum recurvirostre	bma	.	.	.	.	30	.	50
Bryum pseudotriquetrum	bma	.	.	.	.	20	.	30

Nondiagnostic taxa occurring in only one cluster:

Pinus sibirica	tne	40	.	.	.	.	.	.
Polytrichum longisetum	bma	20	.	.	.	.	.	.
Diphasiastrum alpinum	vs	20	.	.	.	.	.	.
Sphagnum fuscum	bms	.	33	.	.	.	.	.
Mylia anomala	bl	.	33	.	.	.	.	.

Calypogeia sphagnicola	bl	.	17	.	.	.	.	.
Drosera rotundifolia	fm	.	17	.	.	.	.	.
Kiaeria blyttii	bma	.	17	.	.	.	.	.
Oxycoccus microcarpus	sde	.	17	.	.	.	.	.
Protothelenella leucothelia	lc	.	17	.	.	.	.	.
Cladonia crispata s. lat.	lfr	.	17	.	.	.	.	.
Icmadophila ericetorum	lc	.	.	27	.	.	.	.
Petasites frigidus	fe	.	.	27	.	.	.	.
Hypogymnia physodes	lfo	.	.	20	.	.	.	.
Arctous alpina	sdd	.	.	20	.	.	.	.
Dicranum groenlandicum	bma	.	.	13	.	.	.	.
Huperzia selago	vs	.	.	13	.	.	.	.
Minuartia arctica	fm	.	.	13	.	.	.	.
Stereocaulon paschale	lfr	.	.	13	.	.	.	.
Varicellaria rhodocarpa	lc	.	.	13	.	.	.	.
Gymnocolea inflata	bl	.	.	13	.	.	.	.
Sphagnum rubellum	bms	.	.	13	.	.	.	.
Diapensia lapponica	fm	.	.	13	.	.	.	.
Sphagnum balticum	bms	.	.	13	.	.	.	.
Sphagnum lenense	bms	.	.	13	.	.	.	.
Sphagnum teres	bms	.	.	7	.	.	.	.
Cynodontium strumiferum	bma	.	.	7	.	.	.	.
Sphagnum warnstorfii	bms	.	.	7	.	.	.	.
Cetraria nigricans	lfr	.	.	7	.	.	.	.
Ochrolechia androgyna	lc	.	.	7	.	.	.	.
Carex rotundata	gs	.	.	7	.	.	.	.
Salix myrtilloides	sld	.	.	7	.	.	.	.
Sphagnum squarrosum	bms	.	.	7	.	.	.	.
Tetralophozia setiformis	bl	.	.	7	.	.	.	.
Peltigera polydactylon	lfo	.	.	7	.	.	.	.
Sphagnum majus	bms	.	.	7	.	.	.	.
Deschampsia sukatschewii	gg	.	.	.	15	.	.	.
Cladonia decorticata	lfr	.	.	.	10	.	.	.
Plagiomnium ellipticum	bma	.	.	.	10	.	.	.
Protomicarea limosa	lc	.	.	.	10	.	.	.
Dicranella subulata	bma	.	.	.	10	.	.	.
Cladonia pleurota	lfr	.	.	.	10	.	.	.
Peltigera kristinssonii	lfo	.	.	.	10	.	.	.
Splachnum sphaericum	bma	.	.	.	10	.	.	.
Plagiothecium berggrenianum	bmp	.	.	.	10	.	.	.

Rinodina turfacea	lc	.	.	.	10	.	.	.
Rumex arcticus	fe	.	.	.	10	.	.	.
Cladonia scabriuscula	lfr	.	.	.	5	.	.	.
Japewia tornoënsis	lc	.	.	.	5	.	.	.
Dicranum majus	bma	.	.	.	5	.	.	.
Polemonium acutiflorum	fe	.	.	.	5	.	.	.
Pachypleurum alpinum	fe	.	.	.	5	.	.	.
Parrya nudicaulis	fe	.	.	.	5	.	.	.
Trisetum spicatum	gg	.	.	.	5	.	.	.
Warnstorfia pseudostraminea	bmp	.	.	.	5	.	.	.
Carex aquatilis	gs	.	.	.	5	.	.	.
Abietinella abietina	bmp	.	.	.	5	.	.	.
Rhexophiale rhexoblephara	lc	.	.	.	5	.	.	.
Hypnum subimponens	bmp	.	.	.	5	.	.	.
Cladonia cyanipes	lfr	.	.	.	5	.	.	.
Salix lanata	sld	.	.	.	5	.	.	.
Aplodon wormskjoldii	bma	.	.	.	5	.	.	.
Sticta arctica	lfo	.	.	.	.	30	.	.
Bacidia bagliettoana	lc	.	.	.	.	20	.	.
Micarea incrassata	lc	.	.	.	.	20	.	.
Myurella tenerrima	bmp	.	.	.	.	20	.	.
Meesia uliginosa	bma	.	.	.	.	20	.	.
Hypogymnia subobscura	lfo	.	.	.	.	20	.	.
Arctocetraria nigricascens	lfr	.	.	.	.	10	.	.
Orthothecium strictum	bmp	.	.	.	.	10	.	.
Warnstorfia sarmentosa	bmp	.	.	.	.	10	.	.
Cephalozia bicuspidata	bl	.	.	.	.	10	.	.
Tortella fragilis	bma	.	.	.	.	10	.	.
Splachnum vasculosum	bma	.	.	.	.	10	.	.
Oncophorus compactus	bma	.	.	.	.	10	.	.
Potentilla hyparctica	fm	.	.	.	.	.	20	.
Micranthes tenuis	fm	.	.	.	.	.	10	.
Siphula ceratites	lfr	.	.	.	.	.	10	.
Lecanora geophila	lc	.	.	.	.	.	.	30
Candelariella placodizans	lc	.	.	.	.	.	.	30
Myurella julacea	bmp	.	.	.	.	.	.	30
Racomitrium panschii	bmp	.	.	.	.	.	.	30
Oncophorus virens	bma	.	.	.	.	.	.	30
Lepraria gelida	lc	.	.	.	.	.	.	20
Peltigera venosa	lfo	.	.	.	.	.	.	10

Cerastium arcticum	fm	.	.	.	.	.	.	10
Sanionia nivalis	bmp	.	.	.	.	.	.	10
Hypnum revolutum	bmp	.	.	.	.	.	.	10
Physconia muscigena	lfo	.	.	.	.	.	.	10
Syntrichia ruralis	bma	.	.	.	.	.	.	10
Psilopilum cavifolium	bma	.	.	.	.	.	.	10
