# Supplementary material for: Vegetation on mesic loamy and sandy soils along a 1700‐km maritime Eurasia Arctic Transect
Source: Appl Veg Sci. 2019 Feb 27;22(1):150–67. doi: 10.1111/avsc.12401 (PMC6519894; doi:10.1111/avsc.12401)
Supplement: Supplementary file 1 — Appendix S1. Geological setting of the Yamal Peninsula. Appendix S2. Typical plot layout. Appendix S3. Eurasia Arctic Transect location and site descriptions. Appendix S4. Eurasia Arctic Transect species cover‐abundance data. Appendix S5. Eurasia Arctic Transect environmental data. Appendix S6. Full synoptic table. Appendix S7. Diagnostic, constant, and dominant taxa for EAT clusters. Appendix S8. Trends of selected soil and vegetation properties vs. summer warmth index. Appendix S9. Regression equations for trend lines of analysed variables. Appendix S10. Number of species per plot along the Eurasia Arctic Transect. Appendix S11. Correlations between four axes of the DCA ordination and environmental variables. Appendix S12. Lichen‐rich tundra of Hayes Island. [file AVSC-22-150-s001.zip › supinfo/Appendix_S7_DiagnosticConstantDominantTaxa_of_clusters_20190210.pdf]

Supporting Information Appendix S7. Diagnostic (Dg), constant (C), and dominant (Dm) taxa in each numerical cluster (Fig. 3 of main text) used in analysis of the Eurasia Arctic Transect vegetation plot data. Determination of diagnostic, constant, and dominant species was determined at two threshold levels. Bolded species are those with the higher threshold values for diagnostic species (fidelity, phi values) and constant species (frequency occurrence). Threshold fidelity values were: diagnostic species (phi values); 50 (**80**); constant species (% frequency): 40 (**50**); dominant species (% with cover >25%).

Cluster 1

Number of relevés: 5

Diagnostic species: ***Vaccinium myrtillus (C) 100.0, Pinus sylvestris (C) 100.0, Larix sibirica (C) 100.0, Betula pubescens (C) 100.0, Juniperus communis (C) 88.0,*** *Peltigera malacea (C) 75.0, Pleurozium schreberi (C, Dm) 72.6, Peltigera leucophlebia (C) 68.5, Cladonia stellaris (C, Dm) 63.8, Empetrum nigrum (C) 63.1, Vaccinium uliginosum (C) 61.3*

Constant species: ***Vaccinium vitis-idaea 100, Vaccinium uliginosum (Dg) 100, Vaccinium myrtillus (Dg) 100, Rhododendron tomentosum s. tomentosum 100, Pleurozium schreberi (Dg, Dm) 100, Pinus sylvestris (Dg) 100, Peltigera leucophlebia (Dg) 100, Larix sibirica (Dg) 100, Empetrum nigrum (Dg) 100, Cladonia stellaris (Dg, Dm) 100, Cetraria islandica 100, Betula pubescens (Dg) 100, Betula nana 100, Juniperus communis (Dg) 80, Polytrichum commune 60, Peltigera malacea (Dg) 60, Cladonia stygia 60, Cladonia rangiferina 60, Cladonia arbuscula s. lat. 60***

Dominant species: *Cladonia stellaris (Dg, C) 100, Pleurozium schreberi (Dg, C) 40*

Cluster 2

Number of relevés: 6

Diagnostic species: ***Carex globularis (C) 100.0, Rubus chamaemorus (C) 86.0, Andromeda polifolia (C) 86.0,*** *Rhododendron tomentosum s. tomentosum (C, Dm) 51.0*

Constant species: ***Vaccinium vitis-idaea 100, Rhododendron tomentosum s. tomentosum (Dg, Dm) 100, Cladonia stygia (Dm) 100, Cladonia amaurocraea 100, Carex globularis (Dg) 100, Rubus chamaemorus (Dg) 83, Cladonia stellaris (Dm) 83, Andromeda polifolia (Dg) 83, Cetraria laevigata 67,*** *Polytrichum strictum 50, Flavocetraria cucullata 50, Cladonia coccifera s. lat. 50, Betula nana 50*

Dominant species: *Cladonia stellaris (C) 67, Rhododendron tomentosum s. tomentosum (Dg, C) 50, Sphagnum fuscum 17, Cladonia stygia (C) 17*

Cluster 3

Number of relevés: 10

Diagnostic species: ***Stellaria longipes taxon edwardsii (C) 100.0, Phippsia algida (C) 100.0, Papaver dahlianum agg. (P. cornwallisense) (C) 100.0, Lecidea ramulosa (C) 100.0, Cochlearia groenlandica (C) 100.0, Orthothecium chryseum (C) 94.5, Cladonia pocillum (C) 94.5, Cetrariella delisei (C) 89.8, Fulgensia bracteata (C) 88.0, Cerastium nigrescens v. laxum (C) 88.0, Saxifraga cernua (C) 84.8, Draba subcapitata (C) 83.3, Cirriphyllum cirrosum (C) 81.6,*** *Cerastium regelii (C) 75.2, Solorina bispora (C) 75.0, Saxifraga cespitosa (C) 75.0, Encalypta alpina (C) 75.0, Bryum rutilans (C) 75.0, Distichium capillaceum (C) 72.1, Cetraria aculeata (C) 69.7, Pohlia cruda (C) 68.1, Stereocaulon rivulorum (C) 67.9, Saxifraga oppositifolia (C) 67.9, Gowardia arctica (C) 67.9, Cladonia symphycarpia (C) 67.9, Polytrichastrum alpinum (C) 64.5, Bartramia ithyphylla (C) 60.4, Polytrichastrum alpinum v. fragile 60.3, Campylium stellatum v. arcticum 60.3, Callialaria curvicaulis 60.3, Ditrichum flexicaule (C) 59.0, Protopannaria pezizoides 55.9*

Constant species: ***Thamnolia vermicularis 100, Stellaria longipes taxon edwardsii (Dg) 100, Polytrichastrum alpinum (Dg) 100, Phippsia algida (Dg) 100, Papaver dahlianum agg. (P. cornwallisense) (Dg) 100, Orthothecium chryseum (Dg) 100, Lecidea ramulosa (Dg) 100, Flavocetraria cucullata 100, Cochlearia groenlandica (Dg) 100, Cladonia pocillum (Dg) 100, Cetrariella delisei (Dg) 100, Cetraria***

Formatted: Position: Horizontal: Right, Relative to: Margin, Vertical: 0", Relative to: Paragraph, Wrap Around

***islandica* 100, *Draba subcapitata* (Dg) 90, *Saxifraga cernua* (Dg) 80, *Pohlia cruda* (Dg) 80, *Fulgensia bracteata* (Dg) 80, *Distichium capillaceum* (Dg) 80, *Cerastium nigrescens* v. *laxum* (Dg) 80, *Stereocaulon alpinum* 70, *Ditrichum flexicaule* (Dg) 70, *Cirriphyllum cirrosum* (Dg) 70, *Cetraria aculeata* (Dg) 70, *Cerastium regelii* (Dg) 70, *Solorina bispora* (Dg) 60, *Saxifraga cespitosa* (Dg) 60, *Encalypta alpina* (Dg) 60, *Bryum rutilans* (Dg) 60,** *Stereocaulon rivulorum (Dg) 50, Saxifraga oppositifolia (Dg) 50, Gowardia arctica (Dg) 50, Cladonia symphycarpia (Dg) 50, Bryoerythrophyllum recurvirostre 50, Bartramia ithyphylla (Dg) 50*

Dominant species: None

Cluster 4

Number of relevés: 15

Diagnostic species: ***Flavocetraria nivalis* (C) 83.3,** *Salix phylicifolia (C) 72.8, Eriophorum vaginatum (C) 72.1, Pedicularis labradorica (C) 70.3, Asahinea chrysantha 60.3, Pertusaria dactylina (C) 57.7, Cladonia grayi 55.9, Schljakovia kunzeana 54.8, Luzula wahlenbergii 54.8*

Constant species: ***Thamnolia vermicularis* 100, *Ptilidium ciliare* 100, *Ochrolechia frigida* 100, *Flavocetraria cucullata* 100, *Cetraria islandica* 100, *Carex bigelowii* (Dm) 100, *Sphaerophorus globosus* (Dm) 93, *Flavocetraria nivalis* (Dg) 93, *Dactylina arctica* 93, *Cladonia stygia* 93, *Cladonia coccifera* s. lat. 93, *Cladonia arbuscula* s. lat. 93, *Vaccinium vitis-idaea* (Dm) 87, *Eriophorum vaginatum* (Dg) 87, *Dicranum elongatum* (Dm) 87, *Cladonia uncialis* 87, *Calamagrostis holmii* 87, *Aulacomnium turgidum* 87, *Empetrum nigrum* 80, *Cladonia subfurcata* 80, *Cladonia amaurocraea* 80, *Bryocaulon divergens* 80, *Betula nana* (Dm) 80, *Sphenolobus minutus* 73, *Rhododendron tomentosum* s. *tomentosum* 73, *Racomitrium lanuginosum* 73, *Polytrichum strictum* 73, *Vaccinium uliginosum* 67, *Salix phylicifolia* (Dg) 67, *Hylocomium splendens* 67, *Cladonia gracilis* s. lat. 67, *Cladonia rangiferina* 60, *Cladonia bellidiflora* 60, *Pedicularis labradorica* (Dg) 53, *Dicranum spadiceum* 53, *Alectoria ochroleuca* 53,** *Pleurozium schreberi 47, Pertusaria dactylina (Dg) 47*

Dominant species: *Betula nana (C) 20, Sphaerophorus globosus (C) 13, Vaccinium vitis-idaea (C) 7, Dicranum elongatum (C) 7, Carex bigelowii (C) 7*

Cluster 5

Number of relevés: 20

Diagnostic species: *Lophozia ventricosa (C) 68.1, Alopecurus borealis (C) 68.0, Salix reptans (C) 62.2, Eriophorum angustifolium (C) 59.0, Tephroseris atropurpurea (C) 58.7, Peltigera canina 56.2, Arctagrostis latifolia (C) 54.9, Peltigera aphthosa 52.1, Lichenomphalia hudsoniana 51.8*

Constant species: ***Thamnolia vermicularis* 100, *Hylocomium splendens* (Dm) 100, *Flavocetraria cucullata* 100, *Dactylina arctica* 100, *Cetraria islandica* 100, *Carex bigelowii* (Dm) 100, *Aulacomnium turgidum* (Dm) 100, *Sphaerophorus globosus* 95, *Dicranum elongatum* (Dm) 95, *Cladonia gracilis* s. lat. 95, *Calamagrostis holmii* (Dm) 95, *Arctagrostis latifolia* (Dg) 95, *Cladonia coccifera* s. lat. 90, *Cladonia amaurocraea* 90, *Bryocaulon divergens* 85, *Sphenolobus minutus* 80, *Lophozia ventricosa* (Dg) 80, *Cladonia uncialis* 75, *Cladonia arbuscula* s. lat. 75, *Racomitrium lanuginosum* 70, *Dicranum spadiceum* (Dm) 70, *Ochrolechia inaequatula* 65, *Vaccinium vitis-idaea* (Dm) 60, *Ptilidium ciliare* 60, *Peltigera scabrosa* 60, *Luzula confusa* 60, *Gowardia nigricans* 60, *Eriophorum angustifolium* (Dg) 60, *Cladonia subfurcata* 60, *Cladonia stygia* 60, *Alopecurus borealis* (Dg) 60, *Salix reptans* (Dg) 55,** *Salix polaris (Dm) 50, Salix nummularia (Dm) 50, Polytrichum strictum (Dm) 50, Peltigera leucophlebia 50, Cladonia rangiferina 50, Tritomaria quinquedentata 45, Tephroseris atropurpurea (Dg) 45, Pohlia nutans 45, Betula nana (Dm) 45*

Dominant species: *Aulacomnium turgidum (C) 25, Hylocomium splendens (C) 20, Carex bigelowii (C) 20, Salix nummularia (C) 15, Betula nana (C) 10, Vaccinium vitis-idaea (C) 5, Salix polaris (C) 5, Polytrichum strictum (C) 5, Dicranum spadiceum (C) 5, Dicranum fuscescens 5, Dicranum elongatum (C) 5, Calamagrostis holmii (C) 5*

Formatted: Position: Horizontal: Right, Relative to: Margin, Vertical: 0", Relative to: Paragraph, Wrap Around

Cluster 6

Number of relevés: 10

Diagnostic species: ***Blepharostoma trichophyllum (C, Dm) 97.2,*** *Salix polaris (C, Dm) 78.2, Tomentypnum nitens (C) 77.6, Dryas octopetala (C) 66.9, Poa arctica (C) 65.6, Juncus biglumis (C) 62.3, Bryum cyclophyllum 60.3, Stellaria longipes (C) 59.8, Arctagrostis latifolia (C) 59.3, Sphenolobus minutus (C) 51.0*

Constant species: ***Thamnolia vermicularis 100, Sphenolobus minutus (Dg) 100, Sphaerophorus globosus 100, Salix polaris (Dg, Dm) 100, Polytrichum strictum 100, Hylocomium splendens (Dm) 100, Dryas octopetala (Dg) 100, Dicranum elongatum (Dm) 100, Cladonia uncialis 100, Cladonia coccifera s. lat. 100, Cladonia arbuscula s. lat. (Dm) 100, Cladonia amaurocraea 100, Cetraria islandica 100, Carex bigelowii (Dm) 100, Calamagrostis holmii 100, Blepharostoma trichophyllum (Dg, Dm) 100, Aulacomnium turgidum 100, Arctagrostis latifolia (Dg) 100, Tomentypnum nitens (Dg) 90, Ptilidium ciliare 90, Ochrolechia frigida 90, Cladonia rangiferina 90, Cladonia gracilis s. lat. 90, Poa arctica (Dg) 80, Dactylina arctica 80, Sanionia uncinata 70, Dicranum acutifolium 70, Tritomaria quinquedentata 60, Stellaria longipes (Dg) 60, Juncus biglumis (Dg) 60,*** *Racomitrium lanuginosum 50, Parmelia omphalodes s. lat. 50, Oncophorus wahlenbergii 50, Lobaria linita 50, Cladonia subfurcata 50, Bryocaulon divergens 50, Anthelia juratzkana 50*

Dominant species: *Salix polaris (Dg, C) 50, Carex bigelowii (C) 50, Hylocomium splendens (C) 40, Cladonia arbuscula s. lat. (C) 40, Dicranum elongatum (C) 10, Blepharostoma trichophyllum (Dg, C) 10*

Cluster 7

Diagnostic species: ***Pogonatum dentatum (C) 80.1,*** *Oxyria digyna (C) 76.7, Gymnomitrion corallioides (C, Dm) 72.6, Luzula confusa (C) 72.1, Salix nummularia (C, Dm) 70.3, Solorina crocea (C) 67.9, Lloydia serotina (C) 67.9, Polytrichum piliferum (C) 56.3, Pohlia crudoides 54.6, Gowardia nigricans (C) 53.5*

Constant species: ***Thamnolia vermicularis 100, Sphaerophorus globosus 100, Salix nummularia (Dg, Dm) 100, Ochrolechia frigida 100, Luzula confusa (Dg) 100, Gymnomitrion corallioides (Dg, Dm) 100, Bryocaulon divergens 100, Racomitrium lanuginosum (Dm) 90, Gowardia nigricans (Dg) 90, Pogonatum dentatum (Dg) 80, Oxyria digyna (Dg) 80, Polytrichum strictum 70, Cladonia coccifera s. lat. 70, Polytrichastrum alpinum 60, Pedicularis hirsuta 60, Parmelia omphalodes s. lat. 60, Cladonia uncialis 60, Cladonia gracilis s. lat. 60, Cetraria islandica 60, Alectoria ochroleuca 60,*** *Solorina crocea (Dg) 50, Polytrichum piliferum (Dg) 50, Lloydia serotina (Dg) 50, Hylocomium splendens 50, Dryas octopetala 50, Dicranum elongatum 50*

Dominant species: *Gymnomitrion corallioides (Dg, C) 50, Racomitrium lanuginosum (C) 40, Salix nummularia (Dg, C) 30*

Formatted: Position: Horizontal: Right, Relative to: Margin, Vertical: 0", Relative to: Paragraph, Wrap Around
