# Supplementary material for: Vegetation on mesic loamy and sandy soils along a 1700‐km maritime Eurasia Arctic Transect
Source: Appl Veg Sci. 2019 Feb 27;22(1):150–67. doi: 10.1111/avsc.12401 (PMC6519894; doi:10.1111/avsc.12401)
Supplement: Supplementary file 1 — Appendix S1. Geological setting of the Yamal Peninsula. Appendix S2. Typical plot layout. Appendix S3. Eurasia Arctic Transect location and site descriptions. Appendix S4. Eurasia Arctic Transect species cover‐abundance data. Appendix S5. Eurasia Arctic Transect environmental data. Appendix S6. Full synoptic table. Appendix S7. Diagnostic, constant, and dominant taxa for EAT clusters. Appendix S8. Trends of selected soil and vegetation properties vs. summer warmth index. Appendix S9. Regression equations for trend lines of analysed variables. Appendix S10. Number of species per plot along the Eurasia Arctic Transect. Appendix S11. Correlations between four axes of the DCA ordination and environmental variables. Appendix S12. Lichen‐rich tundra of Hayes Island. [file AVSC-22-150-s001.zip › supinfo/Appendix_S8_Trends_Soil_and_veg_properties_vs._SWIg_20190210.pdf]

Supporting Information, Appendix S8. Trends in selected soil and vegetation properties along the summer warmth index (SWI_g) gradient.

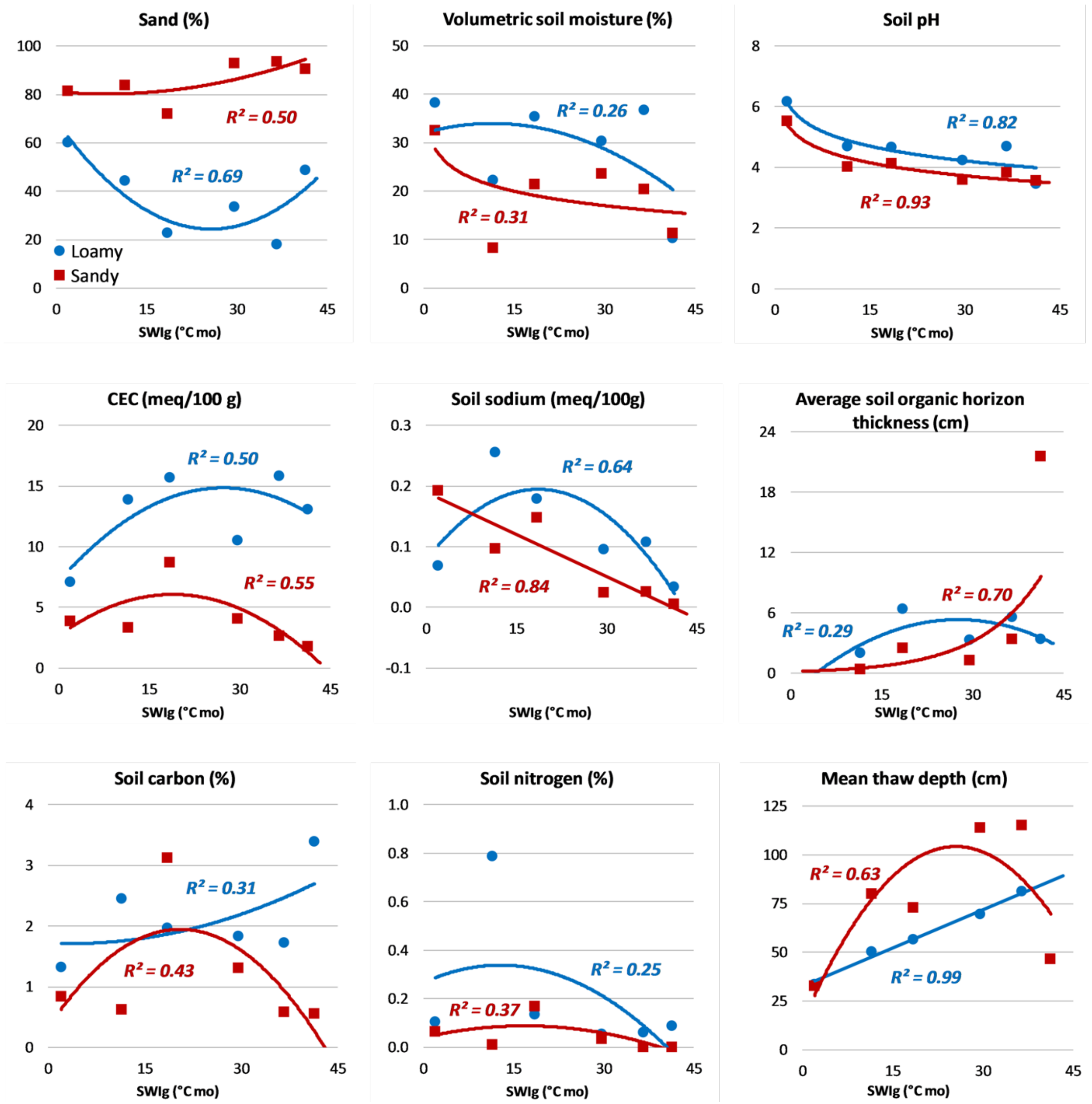

Figure S8.1. Trends in selected soil properties of the top mineral horizon of loamy and sandy sites along the summer-warmth-index (SWI_g) gradient. Variables include percent sand, volumetric soil moisture, soil pH, cation exchange capacity (CEC), soil sodium, thickness of organic soil horizons, percent soil carbon, percent soil nitrogen, and depth of summer thaw at time of measurement. Equations of the trend lines are in Supplemental Information, Appendix S9.

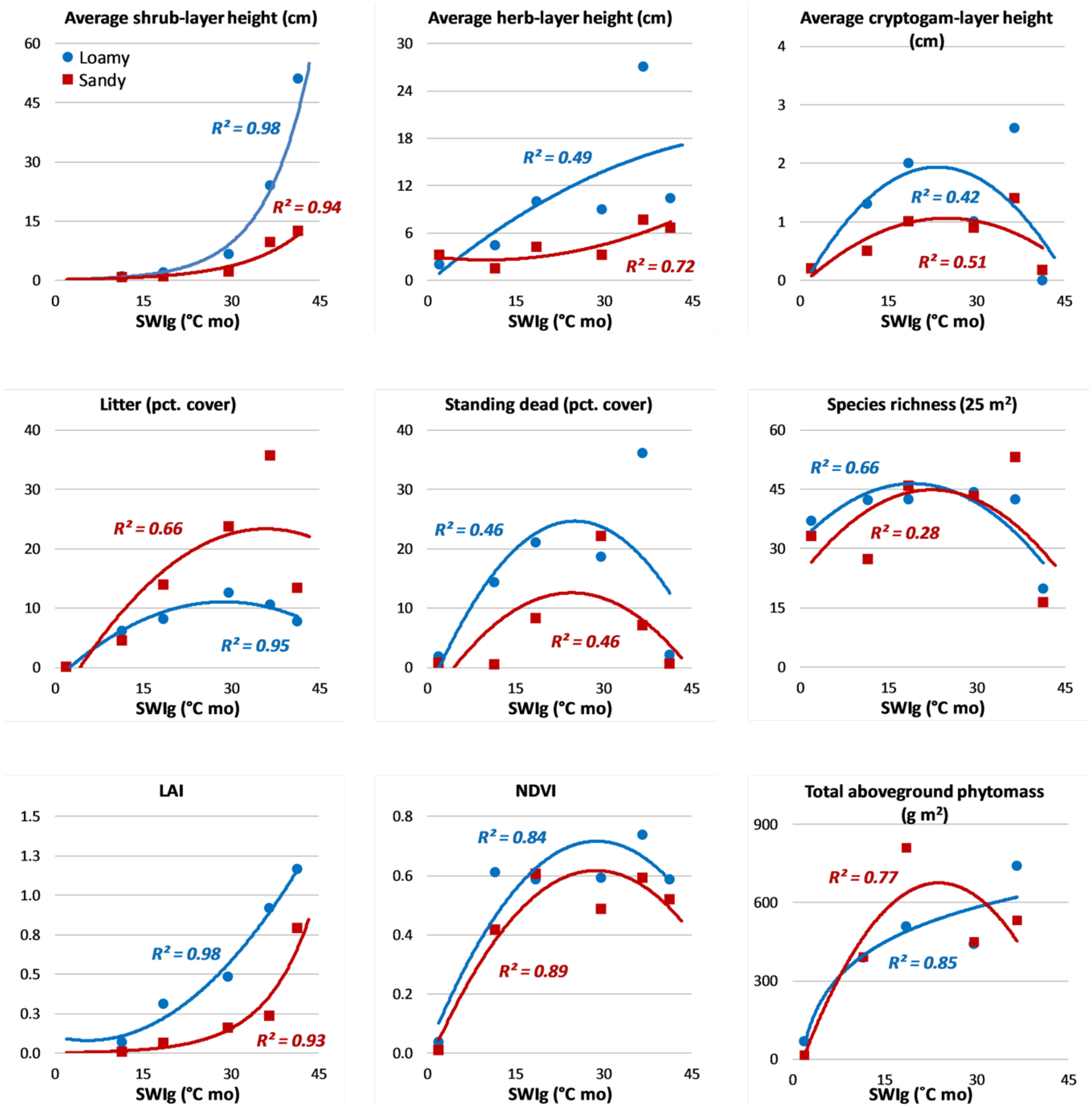

Figure S8.2. Trends of selected vegetation-related factors along the summer-warmth index (SWI_g) gradient on loamy and sandy sites: Shrub-layer height, herb-layer height, moss-layer thickness, live green fraction of total biomass, litter cover, standing dead cover, species richness, leaf area index (LAI), hand-held Normalized Difference Vegetation Index (NDVI). Equations of the trend lines are in Supplemental Information, Appendix S9.
