# Supplementary material for: Vegetation on mesic loamy and sandy soils along a 1700‐km maritime Eurasia Arctic Transect
Source: Appl Veg Sci. 2019 Feb 27;22(1):150–67. doi: 10.1111/avsc.12401 (PMC6519894; doi:10.1111/avsc.12401)
Supplement: Supplementary file 1 — Appendix S1. Geological setting of the Yamal Peninsula. Appendix S2. Typical plot layout. Appendix S3. Eurasia Arctic Transect location and site descriptions. Appendix S4. Eurasia Arctic Transect species cover‐abundance data. Appendix S5. Eurasia Arctic Transect environmental data. Appendix S6. Full synoptic table. Appendix S7. Diagnostic, constant, and dominant taxa for EAT clusters. Appendix S8. Trends of selected soil and vegetation properties vs. summer warmth index. Appendix S9. Regression equations for trend lines of analysed variables. Appendix S10. Number of species per plot along the Eurasia Arctic Transect. Appendix S11. Correlations between four axes of the DCA ordination and environmental variables. Appendix S12. Lichen‐rich tundra of Hayes Island. [file AVSC-22-150-s001.zip › supinfo/Appendix_S9_RegressionEquations_20190210.pdf]

**Supporting Information, Appendix S9. Best-fit regression equations for trend lines of analyzed variables vs. summer warmth index ($SWI_g$, ˚C mo), loamy and sandy sites along the EAT.** Equations are for best-fit trend lines determined in Microsoft Excel.

| Variable | Loamy | | Sandy | |
|---|---|---|---|---|
| | Equation | $R^2$ | Equation | $R^2$ |
| **Mean soil textures vs. $SWI_g$ (˚C mo) (Fig. 2)** | | | | |
| Clay | $y = -0.02x^2 + 1.01x + 5.43$ | 0.28 | $y = -0.01x^2 + 0.21x + 2.33$ | 0.47 |
| Silt | $y = -0.048x^2 + 2.46x + 25.52$ | 0.80 | $y = -0.01x^2 - 0.002x + 16.58$ | 0.53 |
| Sand | $y = 0.07x^2 - 3.48x + 69.05$ | 0.69 | $y = 0.01x^2 - 0.21x + 81.10$ | 0.50 |
| **Cover (pct.) vs. $SWI_g$ (˚C mo) (Fig. 4a–4c)** | | | | |
| Deciduous shrubs | $y = -0.004x^2 + 1.16x - 0.36$ | 0.91 | $y = -0.06x^2 + 2.88x - 4.57$ | 0.38 |
| Evergreen shrubs | $y = 0.01x^2 + 0.11x - 0.05$ | 0.89 | $y = 0.01x^2 + 0.32x - 1.23$ | 0.62 |
| Graminoids | $y = -0.09x^2 + 4.13x - 8.70$ | 0.93 | y = -0.03x2 + 1.49x - 4.53 | 0.53 |
| Forbs | $y = 0.02x^2 - 1.34x + 17.21$ | 0.83 | $y = 0.05x^2 - 2.76x + 31.39$ | 0.91 |
| Bryophytes | $y = -0.13x^2 + 5.89x + 1.84$ | 0.61 | $y = -0.08x^2 + 3.35x + 10.19$ | 0.33 |
| Lichens | $y = 0.18x^2 - 8.80x + 110.23$ | 0.85 | $y = 0.16x^2 - 7.13x + 102.24$ | 0.67 |
| **Mean species richness vs. $SWI_g$ (˚C mo) (Fig. 4d)** | | | | |
| Total species richness | $y = -0.05x^2 + 1.78x + 34.77$ | 0.69 | $y = -0.02x^2 + 1.07x + 30.45$ | 0.68 |
| Deciduous shrubs | $y = 1.18\ln(x) - 1.18$ | 0.82 | $y = 1.04\ln(x) - 1.18$ | 0.52 |
| Evergreen shrubs | $y = 0.08x - 0.45$ | 0.80 | $y = 0.09x - 0.28$ | 0.92 |
| Graminoids | $y = -0.02x^2 + 0.65x - 0.32$ | 0.95 | $y = -0.01x^2 + 0.53x - 0.55$ | 0.66 |
| Forbs | $y = 0.01x^2 - 0.43x + 8.00$ | 0.70 | $y = 0.005x^2 - 0.35x + 7.56$ | 0.91 |
| Bryophytes | $y = -0.02x^2 + 0.46x + 13.45$ | 0.48 | $y = 0.002x^2 - 0.02x + 11.20$ | 0.52 |
| Lichens | $y = -0.03x^2 + 1.10x + 13.25$ | 0.89 | $y = -0.01x^2 + 0.86x + 12.00$ | 0.82 |
| **Soil factors vs. $SWI_g$ (˚C mo) (Supplemental Information, Appendix S8, Fig. S8.1)** | | | | |
| Sand (%) | $y = 0.07x^2 - 3.48x + 69.07$ | 0.69 | $y = 0.01x^2 - 0.22x + 81.12$ | 0.50 |
| Volumetric soil moisture (%) | $y = -0.02x^2 + 0.35x + 31.95$ | 0.26 | $y = -4.30\ln(x) + 31.63$ | 0.31 |
| Soil pH | $y = -0.70\ln(x) + 6.60$ | 0.82 | $y = -0.62\ln(x) + 5.83$ | 0.93 |
| CEC (meq/100 g) | $y = -0.01x^2 + 0.57x + 7.12$ | 0.50 | $y = -0.01x^2 + 0.36x + 2.66$ | 0.55 |
| Soil sodium (meq/100g) | $y = -0.0003x^2 + 0.01x + 0.08$ | 0.64 | $y = -0.005x + 0.19$ | 0.84 |
| Average soil organic horizon thickness (cm) | $y = -0.01x^2 + 0.54x - 2.07$ | 0.29 | $y = 0.17e^{0.10x}$ | 0.70 |
| Soil carbon (%) | $y = 0.001x^2 - 0.005x + 1.72$ | 0.31 | $y = -0.004x^2 + 0.16x + 0.33$ | 0.43 |
| Soil nitrogen (%) | $y = -0.0004x^2 + 0.01x + 0.27$ | 0.25 | $y = -0.0002x^2 + 0.01x + 0.04$ | 0.37 |
| Mean thaw depth (cm) | $y = 1.31x + 32.51$ | 0.99 | $y = -0.14x^2 + 7.06x + 14.20$ | 0.63 |
| **Vegetation factors vs. SWIg (˚C mo) (Supplemental Information, Appendix S8, Fig. S8.2)** | | | | |
| Average shrub-layer height | $y = 0.19e^{0.13x}$ | 0.98 | $y = 0.18e^{0.10x}$ | 0.94 |
| Average herb-layer height | $y = -0.01x^2 + 0.63x - 0.34$ | 0.49 | $y = 0.00x^2 - 0.10x + 3.07$ | 0.72 |
| Average cryptogam-layer height | $y = -0.00x^2 + 0.18x - 0.20$ | 0.42 | $y = -0.00x^2 + 0.09x - 0.11$ | 0.51 |
| Litter (pct. cover) | $y = -0.02x^2 + 0.92x - 2.20$ | 0.95 | $y = -0.02x^2 + 1.68x - 6.74$ | 0.66 |
| Standing dead (pct. cover) | $y = -0.05x^2 + 2.33x - 4.61$ | 0.46 | $y = -0.03x^2 + 1.54x - 6.36$ | 0.46 |
| Species richness (25 $m^2$) | $y = -0.04x^2 + 1.55x + 31.66$ | 0.66 | $y = -0.04x^2 + 1.97x + 22.85$ | 0.28 |
| LAI | $y = 0.001x^2 - 0.01x + 0.11$ | 0.98 | $y = 0.003e^{0.13x}$ | 0.93 |
| NDVI | $y = -0.001x^2 + 0.05x + 0.01$ | 0.84 | $y = -0.001x^2 + 0.05x - 0.04$ | 0.89 |
| Total aboveground phytomass (g $m^2$) | $y = 193.79\ln(x) - 76.71$ | 0.85 | $y = -1.38x^2 + 65.72x - 108.24$ | 0.77 |
